# Ultrasound-Assisted Ternary Deep Eutectic Solvent Extraction of Total Flavonoids from *Artemisia argyi*: GA-ANN Optimization, Greenness Assessment, and In Vitro Bioactivity Evaluation

**DOI:** 10.3390/antiox15091069

**Published:** 2026-08-26

**Authors:** Xuxiang Zhang, Jiafei Long, Zhijia Wang, Yuping Zhang, Tonghao Yang, Yongmei Jiang, Faming Wu, Xin Zhang, Xuqiang Nie, Gang Wang, Sha Liu

**Affiliations:** 1 College of Pharmacy, Zunyi Medical University, Zunyi 563006, China; 2 Guizhou Key Laboratory of Modern Traditional Chinese Medicine Creation, Zunyi Medical University, Zunyi 563006, China; 3 Key Lab of the Basic Pharmacology of the Ministry of Education and Joint International Research Laboratory of Ethnomedicine of Ministry of Education, Zunyi Medical University, Zunyi 563006, China; 4 Zunyi Center for Disease Control and Prevention, Zunyi 563006, China

**Keywords:** *Artemisia argyi*, GA-ANN, greenness, deep eutectic solvent, total flavonoids, antioxidant

## Abstract

Total flavonoids (TF) from *Artemisia argyi*, a traditional edible-medicinal Asteraceae herb, were extracted via a food-grade ternary deep eutectic solvent (TDES, Glycerol/Levulinic Acid/Xylitol = 1:1:1 molar ratio, 30% *w*/*w* water) under ultrasound assistance. A hybrid response surface methodology–genetic algorithm–artificial neural network (RSM-GA-ANN) model optimized parameters to deliver a maximum TF yield of 107.7 mg/g—1.5–1.8-fold higher than conventional hydroalcoholic extraction. Greenness was quantified by AGREEprep (score = 0.70) and MoGAPI (score = 80/100), confirming the method’s sustainability, with TDES retaining >84% extraction efficiency over three reuse cycles. Molecular dynamics simulations revealed TDES forms a more stable hydrogen bond network with plant cell walls (average H-bond lifetime: 101.279 ps vs. 46.698 ps for 50% ethanol), a finding validated by density functional theory calculations showing TDES establishes 7–9 hydrogen bonds with cellobiose (the cellulose repeating unit), far exceeding ethanol’s 1–2 hydrogen bonds. Purified TF exhibited potent radical-scavenging activity (DPPH IC_50_: 0.176 mg/mL; ABTS IC_50_: 0.159 mg/mL) and multipotent enzyme inhibition (α-glucosidase IC_50_: 55.31 μg/mL; acetylcholinesterase IC_50_: 0.618 mg/mL; pancreatic lipase IC_50_: 0.125 mg/mL). TF also suppressed HCT116, A549, and HepG2 proliferation (IC_50_ ≈ 50 μg/mL) and protected HepG2 cells against H_2_O_2_-induced oxidative damage. As an in silico probe, the predominant quantified flavonoid eupatilin (3.64 mg/g) docked to xanthine oxidase (−7.78 kcal/mol vs. allopurinol −6.86), offering a structural hypothesis for XO interaction without attributing mixture bioactivity to a single compound. This TDES-based platform offers a scalable, green route to valorize *A. argyi* for functional food and nutraceutical applications.

## 1. Introduction

The demand for natural antioxidants in food, pharmaceutical, and cosmetic industries has driven intensive research into underutilized botanical resources [1]. *Artemisia argyi* (*A. argyi*), a perennial Asteraceae herb widely distributed across Asia and Europe, is traditionally consumed as young shoots in seasonal foods and used in moxibustion therapy [2]. The leaves of *A. argyi* are abundant in bioactive compounds, mainly including flavonoids like eupatilin and isoscutellarein, as well as polysaccharides, volatile oils and organic acids. These natural ingredients possess multiple pharmacological benefits, such as anti-inflammation, antioxidant capacity, lipid oxidation suppression, and immune regulation [3,4]. Despite this, the high-value conversion of *A. argyi* biomass remains limited, partly due to inefficient extraction technologies.

Flavonoids are among the major antioxidant components of *A. argyi* [5], but traditional extraction methods rely on organic solvents such as methanol and ethanol. These solvents are volatile, toxic, and environmentally harmful, and often yield low amounts of target compounds [6,7]. Deep eutectic solvents (DES) have emerged as green alternatives with low vapor pressure, tunable polarity, and biodegradability [8,9]. Ternary DES (TDES), incorporating a third component into binary DES, further modulate viscosity, hydrogen bonding networks, and selectivity through synergistic interactions [10]. Although TDES have been applied to flavonoid recovery from several botanicals, food-grade TDES for *A. argyi* remain unexplored, and no study has integrated greenness metrics, atomic-scale mechanisms, and multi-bioactivity profiling in a single workflow.

Based on this research background, this study developed a food-grade TDES using glycerol, levulinic acid, and xylitol, aiming to achieve green and efficient extraction of TF from *A. argyi*. We adopted response surface methodology (RSM) together with the GA-ANN hybrid algorithm to optimize the extraction conditions. Meanwhile, scanning electron microscopy (SEM) and molecular dynamics (MD) simulation were applied to reveal the underlying extraction mechanism. Two green evaluation tools, AGREEprep and MOGAPI, were further used to comprehensively assess the environmental friendliness and practical applicability of the established extraction process. In addition, we tested multiple biological functions of the obtained extracts, including in vitro antioxidant capacity, α-glucosidase inhibitory activity for hypoglycemic effect, pancreatic lipase inhibition for anti-obesity potential, as well as acetylcholinesterase inhibition related to neuroprotection. Cell experiments were also carried out to confirm their protective ability against cell damage caused by oxidative stress. Moreover, molecular docking was performed to characterize the atomic-level binding interaction of eupatilin for preliminary mechanistic exploration. To address these gaps, the present study integrates green TDES-ultrasound extraction, multi-bioactivity screening (antioxidant, enzyme-inhibitory, and anti-proliferative), and molecular docking analysis, aiming to establish a comprehensive foundation for the sustainable development and high-value utilization of *A. argyi* resources.

## 2. Materials and Methods

### 2.1. Materials and Reagents

The dried leaves of *Artemisia argyi* Levl. were purchased from Qichai Store, Huanggang, China, and authenticated by Prof. Yujin Zhang (School of Pharmacy, Zunyi Medical University). A voucher specimen (No. CRPV-20251217; accession No. ZMUH-2025-1217) was deposited at Laboratory 2-611, School of Pharmacy, Zunyi Medical University. *A. argyi* is not a rare, threatened, or endangered species, and collection complied with institutional and national guidelines. Human colorectal cancer cell line HCT116 (Cat. No. SNL-077) was purchased from Wuhan Sunncell Biotechnology Co., Ltd., Wuhan, China. Human hepatocellular carcinoma cell line HepG2 (BC-C-HU-024) and human lung cancer cell line A549 (BC-C-HU-009) were purchased from Nanjing Sheng Hang Biotechnology Co., Ltd. (Bio-Channel, Nanjing, China). All cell lines were authenticated by STR profiling and confirmed mycoplasma-free by the suppliers prior to delivery.

### 2.2. Determination of TF from A. argyi

TF from *A. argyi* was extracted using TDES (Gly-LevA-Xyl) via ultrasound (30 mL/g, 320 W, 60 °C, 50 min). TF content was determined by a modified NaNO_2_-Al(NO_3_)_3_-NaOH colorimetric method with quercetin as standard (λ = 510 nm) [11]. The linear regression equation was y = 0.0167x + 0.1509 (R^2^ = 0.9992, 5–40 μg/mL). TDES served as blank control (Supplementary Materials). In addition, a quantitative HPLC-DAD method was developed and validated to simultaneously determine the four major flavonoids (naringin, luteolin, kaempferol, and eupatilin) in the optimized TDES extract. Detailed chromatographic conditions, method validation parameters (calibration curves, LOD, LOQ, precision, stability, repeatability, and recovery), and quantitative results are provided in the Supplementary Materials (Tables S1 and S2, Figure S1).

(1) $$\mathrm{TF}\;\mathrm{extraction}\;\mathrm{yield}\;(\mathrm{mg}/g)=\frac{\mathrm{Mass}\;\mathrm{of}\;\mathrm{TF}\;(\mathrm{mg})}{\mathrm{Mass}\;\mathrm{of}\;\mathrm{crude}\;\mathrm{powder}\;(g)}$$

### 2.3. Preparation and Screening of TDES

Eight BDESs and seventeen TDESs were prepared via heating–stirring (Table S3) [12]. The optimal TDES (Gly-LevA-Xyl) was screened through ultrasound-assisted extraction (50 °C, 30:1 mL/g, 320 W, 40 min) and subsequently optimized by varying molar ratios (1:1:1 to 1:3:1) and water contents (10–50%). The detailed experimental procedure for [brief description of what Section 2.3 contains, e.g., the NaNO_2_–Al(NO_3_)_3_–NaOH colorimetric assay for total flavonoid determination/or other specific content] is provided in the Supplementary Materials.

### 2.4. Structural Characteristics of the TDES

FT-IR characterization was performed as previously described [13], with minor modifications. Briefly, spectra of Gly, LevA, Xyl, and the prepared TDES were acquired using a Shimadzu IRTracer-100 Fourier transform infrared spectrophotometer (Shimadzu Corporation, Kyoto, Japan) equipped with a diamond ATR accessory (32 scans, 4 cm^−1^ resolution, 4000–400 cm^−1^ range) [13,14].

### 2.5. Single Factor Experimental Design

Single-factor experiments were conducted to optimize the extraction of TF from *A. argyi* using a TDES composed of Gly, LevA, and Xyl (1:1:1, mol ratio) with 30% (*w*/*w*) water content. The effects of time (20–70 min), temperature (20–70 °C), liquid-to-solid ratio (10–60 mL/g), and ultrasonic power (200–400 W) on extraction yield were investigated while maintaining all other parameters constant.

### 2.6. Response Surface Methodology and Experimental Design

In this study, we used the Box–Behnken design to optimize the extraction of TF from *A. argyi*. Three key parameters were investigated: liquid-solid ratio (20, 30, 40 mL/g), extraction time (40, 50, 60 min), and extraction temperature (50, 60, 70 °C). Experimental design and statistical analysis were carried out using Design-Expert 13 software (Stat-Ease, Inc., Minneapolis, MN, USA). The variable coded levels are summarized in Table S4, and the corresponding TF extraction yields are listed in Table S5.

### 2.7. GA-ANN Hybrid Modeling Approach

A backpropagation (BP) neural network (3 inputs, 8 hidden neurons, 1 output) was constructed in MATLAB (R2023b, MathWorks, Natick, MA, USA) using the Levenberg–Marquardt algorithm, with TF yield as the output. To avoid potential overfitting and rigorously evaluate the generalization capability of the GA-BP neural network, the 17 experimental data points derived from the RSM were randomly divided into three distinct subsets: a training set (12 data points, ~70%), a validation set (3 data points, ~18%), and an independent test set (2 data points, ~12%). The training set was used to update network weights and biases, the validation set was employed to monitor for overfitting and fine-tune parameters during training, and the independent test set was reserved to provide an unbiased evaluation of the final model’s predictive performance. Input and output data were normalized to the [0, 1] interval using the mapminmax function prior to training. The trained ANN served as the fitness function for GA optimization (population: 50, generations: 100) to maximize TF yield within defined variable ranges, and optimal extraction conditions were determined (see Supplementary Materials for ANN performance plots, including regression fits, error histograms, MSE convergence curves, and GA fitness evolution) [13,15].

### 2.8. Comparison of TDES Ultrasonic Extraction with Other Methods

To further verify the superiority of the TDES ultrasonic extraction system in extracting the TF content of *A. argyi* in this study, we chose to compare it with the traditional solvent system (50% methanol, 70% methanol, 50% ethanol, 70% ethanol). The ultrasonic extraction of TDES and the remaining residue after traditional method extraction were subjected to SEM characterization studies.

### 2.9. TDES System and Recovery of TF from A. argyi

Nine macroporous resins (AB-8, HPD-100, D101, NKA-2, HPD-750, ADS-17, XAD-7HP, ADS-8 and FPX-66) were screened for static adsorption and desorption performance. XAD-7HP was selected as the optimal resin based on adsorption and desorption rates. Static adsorption kinetics were investigated over 0–180 min. Desorption efficiency was evaluated using 50%, 70%, and 100% methanol and ethanol solutions. TDES recycling performance was assessed by repeating the extraction cycle with recovered solvent until the relative extraction yield fell below 80% (see Sections S1.5.1–S1.9.4 in the Supplementary Materials) [16,17].

### 2.10. Molecular Dynamics (MD) Simulations

Initial 3D molecular structures of eupatilin and solvent components (glycerol/levulinic acid/xylitol and 30% water) were constructed and geometrically optimized using Discovery Studio 2024. Restrained electrostatic potential (RESP) charges were fitted with Multiwfn (v3.8) based on quantum-chemical single-point calculations, and topology files with bonded and non-bonded parameters were generated under the GAFF2 force field using Sobtop (v1.0). Each simulation system was solvated in a cubic box (≈7 × 7 × 7 nm^3^) with TIP3P water, and energy minimization was performed using the steepest-descent algorithm (max 50,000 steps, emtol = 1000 kJ·mol^−1^·nm^−1^). Pre-equilibration consisted of 100 ps NVT (V-rescale thermostat, τ = 0.1 ps, 298.15 K) followed by 100 ps NPT (Parrinello–Rahman barostat, τ = 2 ps, 1 bar), with all bonds involving hydrogen constrained by LINCS and long-range electrostatics handled by PME (real-space cutoff = 0.9 nm). Production MD was carried out for 20 ns in the NPT ensemble (2 fs timestep, LINCS constraints, coordinates saved every 10 ps). Hydrogen bond analysis was performed using GROMACS gmx hbond with a donor–acceptor distance ≤ 3.5 Å and a donor–hydrogen–acceptor angle ≥ 120°; average H-bond lifetime (τ) was derived by fitting the continuous lifetime distribution to a single-exponential decay function C(t) = exp(−t/τ) over three independent repeats. Full details of system preparation, equilibration protocols, production settings, and analysis procedures are provided in Section S1.6 of the Supplementary Materials [18,19].

### 2.11. Quantum Chemical Calculations (DFT)

All quantum chemical simulations were executed via the ORCA 6.1.1 quantum chemistry suite [20] to elucidate the molecular-scale solvation mechanism of the ternary deep eutectic solvent (TDES, Gly-LevA-Xyl) toward cellulose. Cellobiose, the β-1,4-linked disaccharide repeating unit of cellulose and the core structural component of *A. argyi* cell walls, was selected as a representative model compound for polysaccharide-solvent interaction studies. Geometry optimizations and harmonic frequency analyses were performed at the B3LYP [21] density functional level, coupled with Grimme’s D3(BJ) dispersion correction [22,23] to accurately capture van der Waals interactions. The resolution-of-identity Coulomb-fitting (RIJCOSX) approximation [24] was invoked to accelerate computational efficiency. The def2-SVP basis set [25,26] was employed for all structural optimizations, and frequency calculations confirmed the absence of imaginary frequencies, verifying that all optimized geometries corresponded to true energetic minima on the potential energy surface. Bulk aqueous solvation effects (consistent with the 30% water content in experimental TDES systems) were modeled during optimization using the polarizable continuum model (PCM) [27].

For high-precision binding energy evaluation, single-point energy calculations were conducted at the def2-TZVP basis set level [25,26]. Binding energies (ΔE) were defined via the supermolecular approach as ΔE = E(AB) − E(A) − E(B), where E(AB) is the total energy of the solvent-cellobiose complex, and E(A) and E(B) are the energies of isolated solvent monomers and cellobiose, respectively. The solute electron density-based SMD solvation model [28] was applied during single-point calculations to more accurately reflect the dielectric environment of the experimental aqueous TDES system. All molecular visualizations were generated using the VMD program [29].

### 2.12. In Vitro Antioxidation

The antioxidant activities of purified TF were evaluated by DPPH, ABTS, and hydroxyl radical scavenging assays (see Sections S1.7.1–S1.7.3 in the Supplementary Materials). Sample solutions at varying concentrations were reacted with corresponding radical working solutions under standardized conditions (DPPH: 30 min dark incubation, 517 nm; ABTS: 6 min dark incubation, 734 nm; hydroxyl: 37 °C, 30 min, 510 nm). Ascorbic acid served as the positive control [30,31,32].

### 2.13. Antioxidant Molecule Interaction Study

Molecular docking of eupatilin (the predominant flavonoid in *A. argyi* TF) with ABTS and DPPH radicals was performed using AutoDock Vina 1.2.7, with eupatilin as the ligand and the radical molecules as receptors (Supplementary Materials) [33,34,35].

### 2.14. The Protective Effect of TF on Oxidatively Damaged HepG2 Cells

HepG2 cells (1 × 10^4^ cells/well) were incubated for 24 h, and then H_2_O_2_ (0.06 mmol/L) was added for another 4 h to establish the oxidative damage model. The cytoprotective potential of TF against HepG2 cells was examined using a tetrazolium-based colorimetric assay. Cells were preconditioned with varying TF doses (1–6 μg/mL) for 24 h prior to CCK-8 incubation and absorbance measurement at 450 nm. The calculation was performed according to Equation (2) [36,37].

(2) $$Cell\;viability\;(\%)=\frac{A_{s}-A_{b}}{A_{c}-A_{b}}\times 100\%\;$$

where the absorbance values are: sample (*A_s_*), control (*A_c_*), and blank (*A_b_*).

### 2.15. In Vitro Anti-Tumor Experimental Methods

The effects of TF on three types of tumor cells were investigated: colorectal cancer cell line (HCT116), lung cancer cell line (A549), and liver cell carcinoma cell line (HepG2) [38,39]. Cells in the exponential growth phase were inoculated at a density of 5 × 10^4^ cells per well in a 96-well microplate. After 24 h of adherent growth, a series of TF dilution solutions (0–100 μg/mL, logarithmically increasing) were added. The drug effect was observed for 24 h, followed by the determination of absorbance at 450 nm using CCK-8 and calculation of cell survival rate [40].

### 2.16. In Vitro Enzyme Inhibitory Activity

The in vitro bioactivities of purified TF were evaluated through enzyme inhibitory activity assays targeting glucose and lipid metabolism, as well as neuroprotection. Specifically, α-glucosidase, acetylcholinesterase (AChE), and porcine pancreatic lipase inhibitory activities were assessed at concentration ranges of 5–150 μg/mL, 0.25–3 mg/mL, and 0.05–2.0 mg/mL, respectively. Acarbose, tacrine, and simvastatin were used as positive controls in the corresponding tests. The absorbance values were measured at 412 nm, 412 nm, and 405 nm, respectively (Supplementary Materials) [41,42,43].

### 2.17. Molecular Docking Procedure of Eupatilin with Xanthine Oxidase

The crystal structure of xanthine oxidase (XO, PDB ID: 1FIQ) was retrieved from the RCSB Protein Data Bank (https://www.rcsb.org/) [44]. The protein was prepared using AutoDockTools 1.5.6 [45]: all water molecules, ions, and the co-crystallized salicylate ligand were removed; polar hydrogens were added; and Gasteiger charges were assigned. The 3D structure of eupatilin—the predominant flavonoid in the *A. argyi* TDES extract (3.64 ± 0.05 mg/g)—was constructed and geometrically optimized using the MMFF94 force field via OpenBabel 2.3.1 [46], and subsequently converted to pdbqt format. Allopurinol, the clinical XO inhibitor [47], was used as the reference ligand and processed identically.

Molecular docking was performed using AutoDock Vina 1.2.7 [33,48]. The docking grid box was centered at the molybdenum-containing active site (center_x = 26.63, center_y = 10.04, center_z = 113.31 Å), with grid dimensions of 12.80 × 13.92 × 14.35 Å^3^ and a grid spacing of 0.375 Å. The exhaustiveness parameter was set to 32. For each ligand, 10 docking poses were generated, and the conformation with the lowest binding energy (most negative score) was selected for interaction analysis. To validate the docking protocol, the co-crystallized salicylate ligand was re-docked into the active site, and the resulting RMSD was confirmed to be <2.0 Å, indicating the reliability of the docking procedure.

Ligand-protein interactions were analyzed using Discovery Studio Visualizer 2024 (Biovia, San Diego, CA, USA) [49]. Hydrogen bonds were defined as donor–acceptor distances ≤3.5 Å with angles ≥120°. Visualizations of 3D binding conformations were generated using PyMOL 3.0 (Schrödinger, LLC, New York, NY, USA) [50].

### 2.18. Statistical Analysis

All experiments were performed in triplicate (*n* = 3) using three independent extraction replicates. For each condition, approximately 1.0 g of dried *Artemisia argyi* leaf powder from the same homogenized batch was independently weighed three times; each sample underwent a separate ultrasound-assisted extraction, and one measurement (UV-Vis or HPLC) was taken per extract. Data are expressed as mean ± SD (standard deviation). One-way ANOVA followed by Tukey’s honestly significant difference (HSD) post hoc test was used to assess significance; adjusted *p* < 0.05 was considered statistically significant. Groups sharing the same letter (a, b, c) are not significantly different (*p* ≥ 0.05). All analyses were performed using SPSS Statistics 26.0 (IBM Corp., Armonk, NY, USA).

## 3. Results and Discussion

### 3.1. Screening of TDES

**Figure 1:**
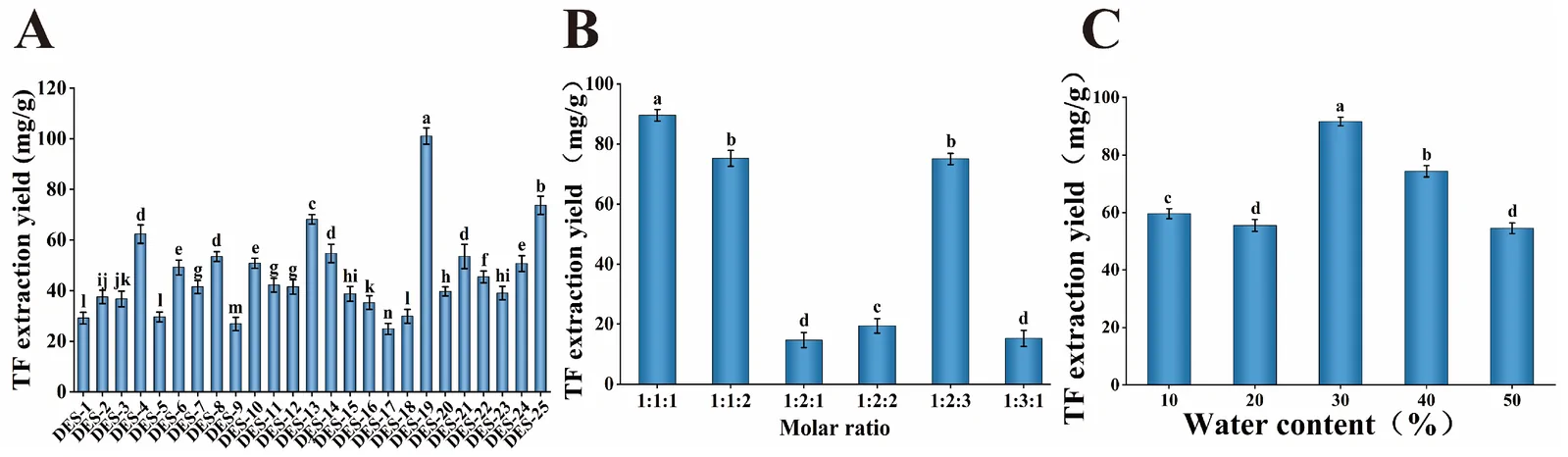
(**A**) filter results for TDES; (**B**) molar ratio of TDES; (**C**) water content of TDES. Different lowercase letters above bars indicate significant differences (*p* < 0.05) according to Tukey’s HSD test.

Twenty-five DESs were screened for TF extraction from *A. argyi*, with Gly-LevA-Xyl (DES-19) achieving the highest yield (101 ± 4.3 mg/g) among 17 TDESs (Figure 1A). The molar ratio and water content were subsequently optimized, with the maximum TF yield obtained at a Gly-LevA-Xyl ratio of 1:1:1 and 30% water content (Figure 1B,C). These conditions were employed for all subsequent extractions. Subsequent quantitative HPLC-DAD analysis of the extract obtained under these optimal conditions revealed that the contents of the four major flavonoids were 1.01 ± 0.01 mg/g for naringin, 1.22 ± 0.02 mg/g for luteolin, 0.390 ± 0.004 mg/g for kaempferol, and 3.64 ± 0.05 mg/g for eupatilin (dry weight basis), confirming eupatilin as the predominant bioactive flavonoid in the TDES extract (see Supplementary Materials for full method validation).

### 3.2. Structural Characterization of TDES

**Figure 2:**
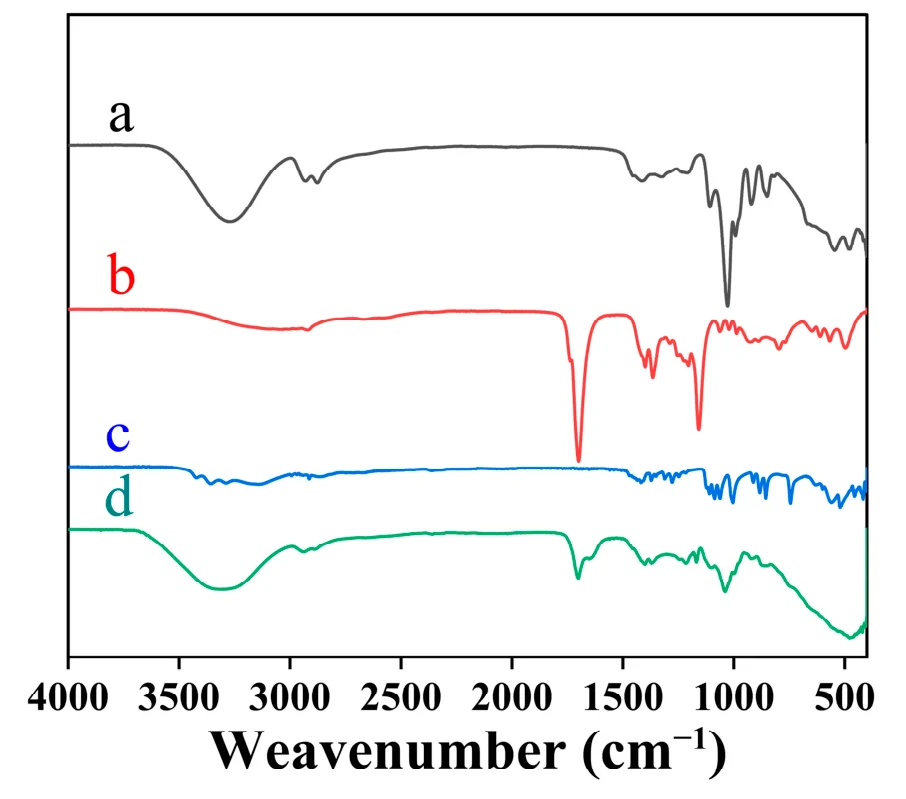
FT-IR spectra of a—Glycerol, b—Xylitol, c—Levulinic Acid and d—TDES.

The formation of the TDES was verified by FT-IR spectroscopy, as shown in Figure 2. Gly displayed typical absorption peaks including O-H stretching (3500–3000 cm^−1^), C-H/O-H bending (1500–1300 cm^−1^), and C-O stretching (1100–1000 cm^−1^). Xyl showed a wide O-H stretching band (3500–3000 cm^−1^), strong C-O stretching near 1000 cm^−1^, and overlapping C-H/O-H bending signals around 1400 cm^−1^. LevA exhibited characteristic carboxylic O-H (3300–2500 cm^−1^), C=O stretching (1750 cm^−1^), and C-O stretching (1000 cm^−1^). In the TDES spectrum, the widened O-H peak (3500–3000 cm^−1^), preserved carboxylic O-H band (2500 cm^−1^), strengthened C=O signal (1750 cm^−1^), and intense C-O vibration (1000 cm^−1^) together suggested the formation of a dense intermolecular hydrogen bonding network among the three components. These observations were consistent with those reported in previous DES studies [12,13,51,52].

### 3.3. Single-Factor Experimental Design

**Figure 3:**
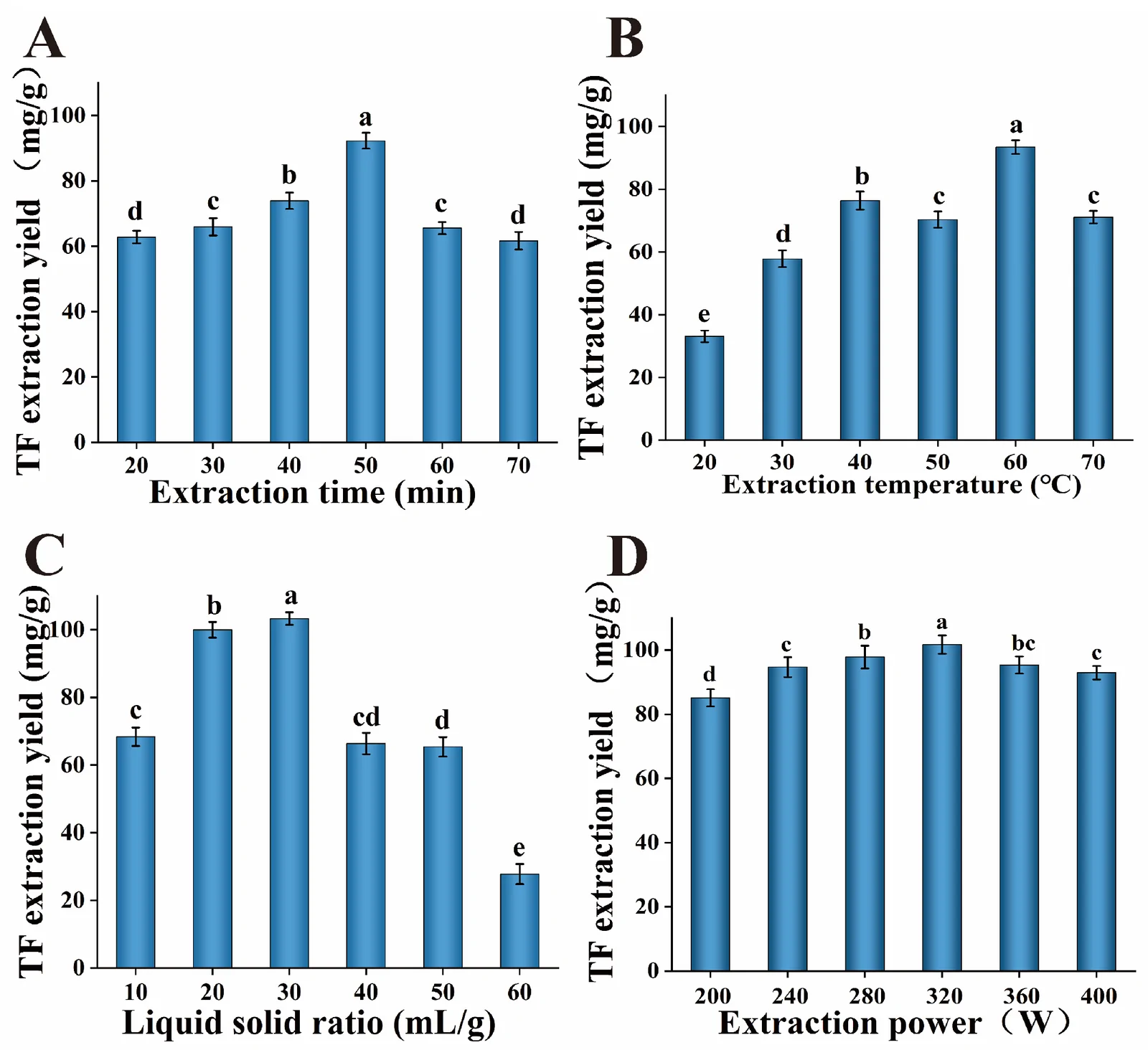
Effects of different factors on the extraction content of TF from *A. argyi* ((**A**) Extraction time; (**B**) Extraction temperature; (**C**) Liquid-solid ratio; (**D**) Extraction power). Different lowercase letters denote significant differences at *p* < 0.05 based on Tukey’s HSD test.

The effects of extraction parameters on TF yield were investigated through single-factor experiments (Figure 3). Extraction yield initially increased with ultrasonic duration, reaching a maximum at 50 min before declining at prolonged times due to potential thermal degradation (Figure 3A). The extraction yield rose gradually as temperature increased from 20 to 60 °C, reaching its highest level at 60 °C. When the temperature continued rising to 70 °C, the yield dropped noticeably, mainly because high heat triggered the degradation of flavonoid compounds (Figure 3B). For the liquid-to-solid ratio, the maximum extraction yield of 103 ± 1.3 mg/g was obtained at 30 mL/g. Further increasing the ratio only led to a dilution effect, which in turn lowered the extraction efficiency (Figure 3C). Ultrasonic power followed a similar changing pattern. The best extraction outcome appeared at 320 W, while higher power levels resulted in a gradual yield decline. This reduction was probably caused by intense cavitation, which damaged and decomposed the target flavonoids (Figure 3D). Consequently, the optimal single-factor conditions were established as 50 min, 60 °C, 30 mL/g and 320 W for extraction time, temperature, liquid-to-solid ratio and ultrasonic power, respectively.

### 3.4. Response Surface Optimization Experiment

The response surface test results are shown in Table S5. The binary multiple regression equation for the TF extraction rate in *A. argyi* was simulated as follows:

Y = 105.3 + 5.30A + 1.08B − 0.475C + 3.65AB − 1.55AC − 3.15BC − 14.6A^2^ − 8.75B^2^ − 9.69C^2^

To validate the constructed binary multiple regression model, analysis of variance (ANOVA) was employed to test the statistical significance of the model and its regression coefficients. The results are shown in Table S6. This model has *R*^2^ = 0.9744 and *R*^2^*Adj* = 0.9416, indicating that the model can accurately reflect the relationship between the response value and the variables. The F-value of this complete model is 29.64, *p* < 0.0005, and the residual term is 0.1672 > 0.05, indicating that the model is reliable, the data are normal, and it can accurately analyze the TF content in *A. argyi*.

**Figure 4:**
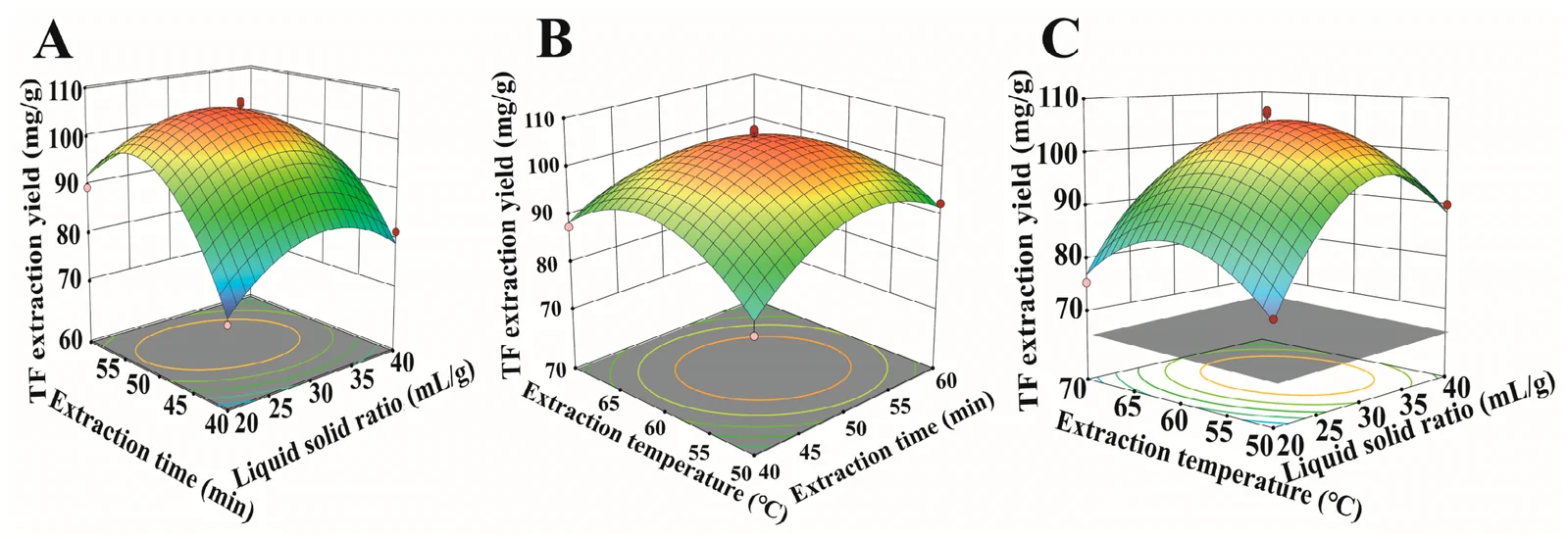
3D Response Surfaces of TF. ((**A**) Extraction time vs. liquid-solid ratio; (**B**) Extraction time vs. temperature; (**C**) Liquid-solid ratio vs. extraction temperature). Color gradient represents the magnitude of TF extraction yield; contour lines on the bottom plane denote the response values.

The 3D response surface plots clearly reflect the interactive effects of different experimental factors on TF extraction yield. As presented in Figure 4, all plotted surfaces displayed an upward concave shape. This trend indicated that any two interacting parameters exerted a combined effect on flavonoid extraction efficiency, which initially increased and then decreased to reach a peak value. Such variation was consistent with the findings from single-factor tests, further verifying the reliability and rationality of the established prediction model.

The model predicted the optimal theoretical parameters for flavonoid extraction from *A. argyi* as follows: extraction time 46.6 min, extraction temperature 58.0 °C, and liquid–solid ratio 24.2 mL/g. Under these ideal conditions, the predicted flavonoid yield was 98.6 mg/g. To accommodate actual experimental operation feasibility, the theoretical parameters were appropriately rounded and revised to 47 min, 58 °C, 25 mL/g, together with a fixed ultrasonic power of 320 W. Validation experiments under these modified practical conditions yielded a higher total flavonoid content of 105 ± 1.4 mg/g.

### 3.5. GA-ANN Optimization

**Figure 5:**
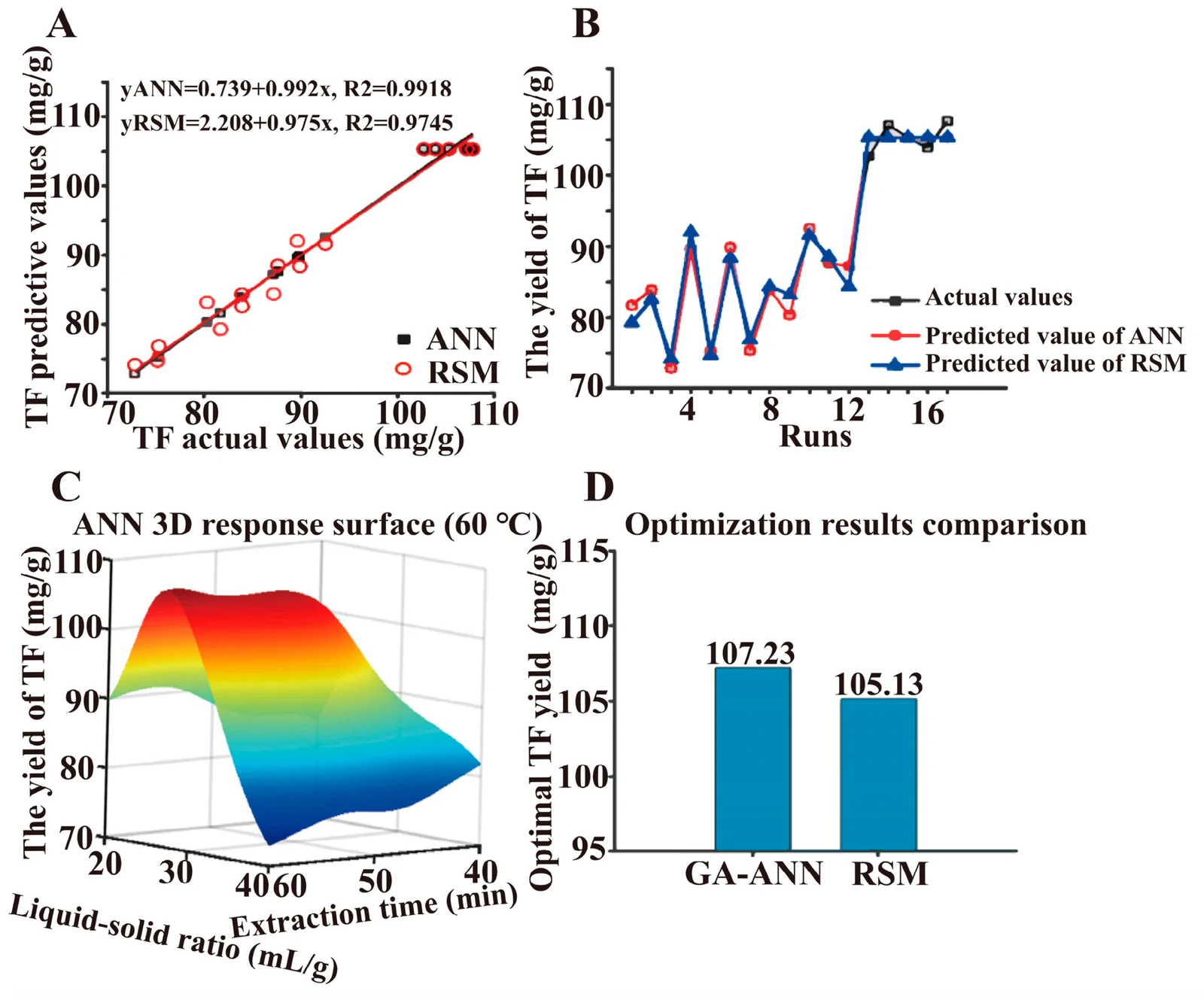
Performance and optimization comparison of ANN and RSM models. (**A**) Correlation between measured and predicted TF yields; (**B**) Predicted versus actual yields for 17 experimental trials; (**C**) ANN three-dimensional response surface showing the combined effects of liquid–solid ratio and extraction time on total flavonoid yield at 60 °C; (**D**) Optimal TF yields determined by GA-ANN and RSM.

The TF extraction process was modeled and optimized using RSM and GA-ANN. A Box–Behnken design established the RSM quadratic polynomial model, while a three-input single-hidden-layer BP-ANN was constructed in MATLAB and coupled with GA for global optimization. Following the dataset partitioning strategy described in Section 2.7, the 17 RSM-derived experimental points were allocated to a training set (12 groups, ~70%), a validation set (3 groups, ~18%), and an independent test set (2 groups, ~12%) to rigorously evaluate model generalization and exclude overfitting. The ANN model exhibited superior predictive accuracy (*R*^2^ = 0.9918 vs. 0.9745) and nonlinear mapping capability compared to RSM (Figure 5A,B). The robustness of this partitioned validation strategy was confirmed by the model’s performance on unseen data. As illustrated in Figures S2 and S3A, the coefficient of determination (*R*^2^) for the training, validation, and independent test sets reached 0.9922, 0.9991, and 1.0000, respectively, indicating excellent generalization. Furthermore, the error histogram (Figure S3B) showed that prediction errors for all datasets were tightly clustered near zero. The MSE convergence curve (Figure S3C) demonstrated synchronous decreases in training and validation errors without divergence, effectively ruling out overfitting. The GA fitness evolution curve (Figure S3D) verified that the optimization process converged stably to the global optimum.

To further quantify the performance difference, root mean square error (RMSE) and mean absolute error (MAE) were calculated for both models. The ANN model yielded an RMSE of 0.87 mg/g and an MAE of 0.62 mg/g, significantly lower than the RSM model’s RMSE (2.1 mg/g) and MAE (1.6 mg/g). This indicates that the ANN model captures the nonlinear coupling effects between extraction parameters more accurately, avoiding the local optimum limitations of the quadratic RSM model. The 3D response surface revealed significant nonlinear coupling effects among variables with a distinct optimal region (Figure 5C). GA-ANN optimization yielded conditions of 33 mL/g, 59 min, and 60 °C with a predicted yield of 107 mg/g, outperforming RSM (25 mL/g, 47 min, 58 °C, 105 mg/g). Experimental verification showed that the GA-ANN hybrid model delivered the best optimization performance. Under the optimized conditions of liquid-to-solid ratio 33 mL/g, extraction time 60 min, and temperature 60 °C, the maximum flavonoid yield reached 108 ± 1.7 mg/g (Figure 5D). The findings further indicated that the GA-ANN method can efficiently avoid falling into local optimal solutions. Compared with other conventional models, this hybrid algorithm is better applicable to the accurate parameter optimization of flavonoid extraction from plant materials [12,53].

### 3.6. Evaluation of Greenness in the Extraction Process

**Figure 6:**
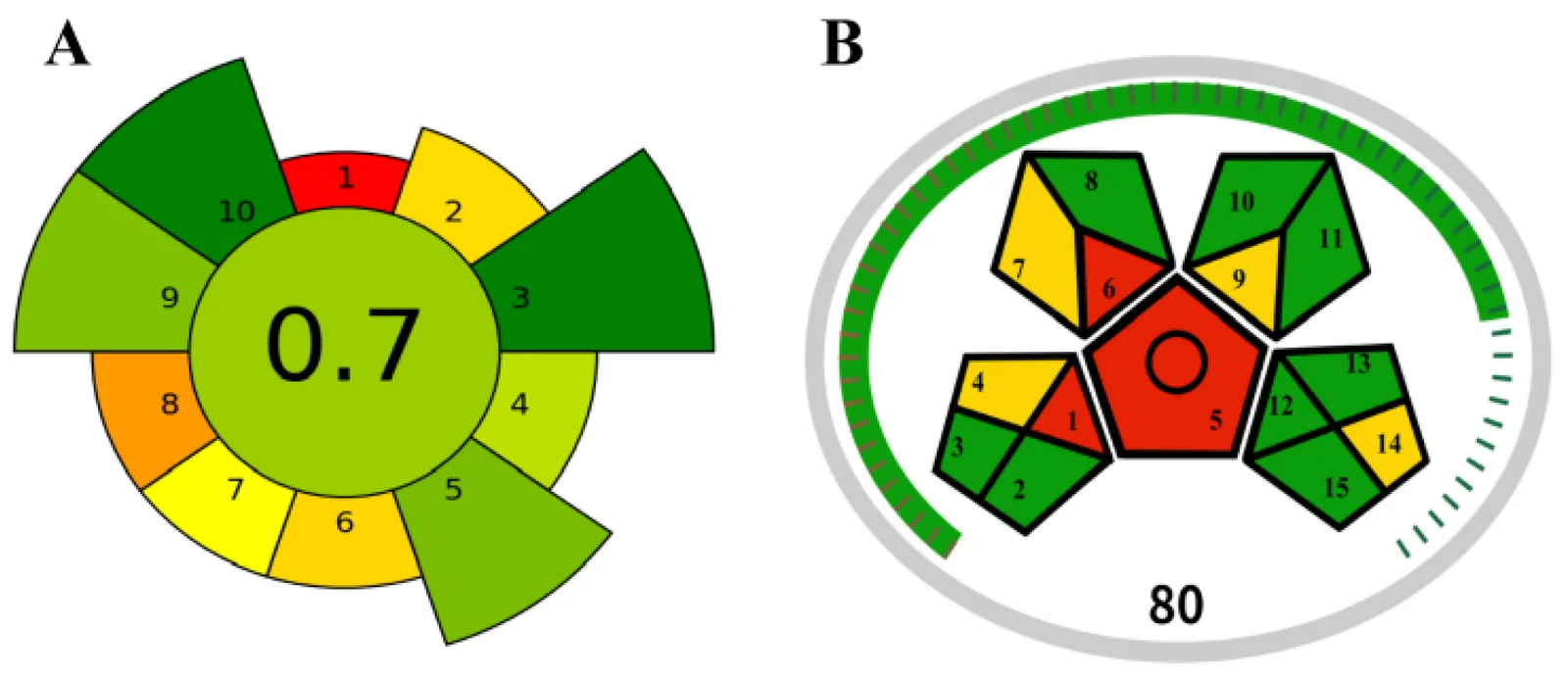
(**A**) AGREEprep and (**B**) MoGAPI score of the extraction process. Different colored segments represent individual evaluation indicators; numbers denote the score of each sub-criterion; the central value corresponds to the overall comprehensive score.

This work adopted two classic green analytical chemistry evaluation tools, namely AGREEprep and MoGAPI, to quantitatively evaluate the environmental performance of the ultrasonic-assisted extraction of TF from *A. argyi*, and the relevant evaluation results are summarized in Figure 6.

As illustrated in Figure 6A, the overall AGREEprep value of the established extraction procedure reached 0.70. As a dedicated evaluation system for the green degree of sample pretreatment protocols, the AGREEprep scale ranges from 0 to 1, with higher scores representing better adherence to green chemistry guidelines [54]. The obtained score of 0.70 revealed that the developed extraction approach exhibits satisfactory performance in multiple green evaluation indicators, such as less consumption of toxic reagents, lower energy input, and improved operational safety, thereby achieving a relatively high eco-friendly level. Such desirable green performance mainly benefits from the reasonable design of the extraction strategy, including the adoption of green TDESs and the rational optimization of experimental parameters. Nevertheless, there is still a certain gap compared with the ideal green analytical protocol with a score close to 1.0. It is thus feasible to further improve the environmental friendliness by screening more eco-compatible solvents or introducing emerging green technical means such as online pretreatment in follow-up research.

The MoGAPI evaluation result in Figure 6B gave a total score of 80 points out of 100. The MoGAPI system can holistically assess the green property, practicability, and comprehensive performance of analytical methodologies [55]. The relatively high score of 80 demonstrated that the proposed extraction method not only meets environmental requirements, but also achieves a good trade-off in core analytical characteristics involving extraction efficiency, selectivity, sensitivity and experimental operability. This finding is consistent with the prevailing development tendency of modern analytical chemistry that pursues simultaneous improvement of greenness and analytical performance [56]. In comparison with conventional extraction techniques documented in previous studies, which generally yield MoGAPI scores between 50 and 70 [57], our method retains reliable analytical quality while greatly improving the green characteristics of the whole workflow, offering a more sustainable alternative for flavonoid extraction.

To further contextualize these results, we benchmarked our scores against recently published DES- or TDES-based extraction methods (2024–2026). Our AGREEprep value of 0.70 falls within the upper-middle range of recently reported DES-based methods, comparable to the AGREE score of 0.75 for switchable DES microextraction and higher than the AGREEprep of 0.50–0.64 reported for other NADES-based extraction systems, though slightly lower than the highest reported value of 0.87 for microwave-assisted NADES extraction, which benefits from the inherent energy efficiency of microwave heating. Our MoGAPI score of 80/100 aligns with the “excellent green” category (>75) and demonstrates that the TDES-ultrasound platform meets stringent green analytical chemistry criteria [56]. Beyond these numerical metrics, the food-grade composition of our TDES and its three-cycle recyclability with >84% efficiency retention provide practical sustainability advantages that reinforce the green credentials of our platform. Combining the assessment outcomes of AGREEprep and MoGAPI, it is evident that the newly developed extraction strategy integrates prominent environmental advantages and stable analytical performance. The high MoGAPI score confirms its good practical application potential, while the AGREEprep evaluation also points out clear directions for further green upgrading. This study can serve as a valuable reference for the optimization of analogous plant extraction workflows, and proves that standardized greenness evaluation tools can effectively guide process modification to promote sustainability without sacrificing analytical accuracy. Subsequent research could target the low-scoring indicators in the AGREEprep assessment, such as developing solvent-free extraction modes and further cutting energy consumption, to further elevate the green level of the whole extraction process.

### 3.7. Comparison of the TDES System with Traditional Extraction Systems

**Figure 7:**
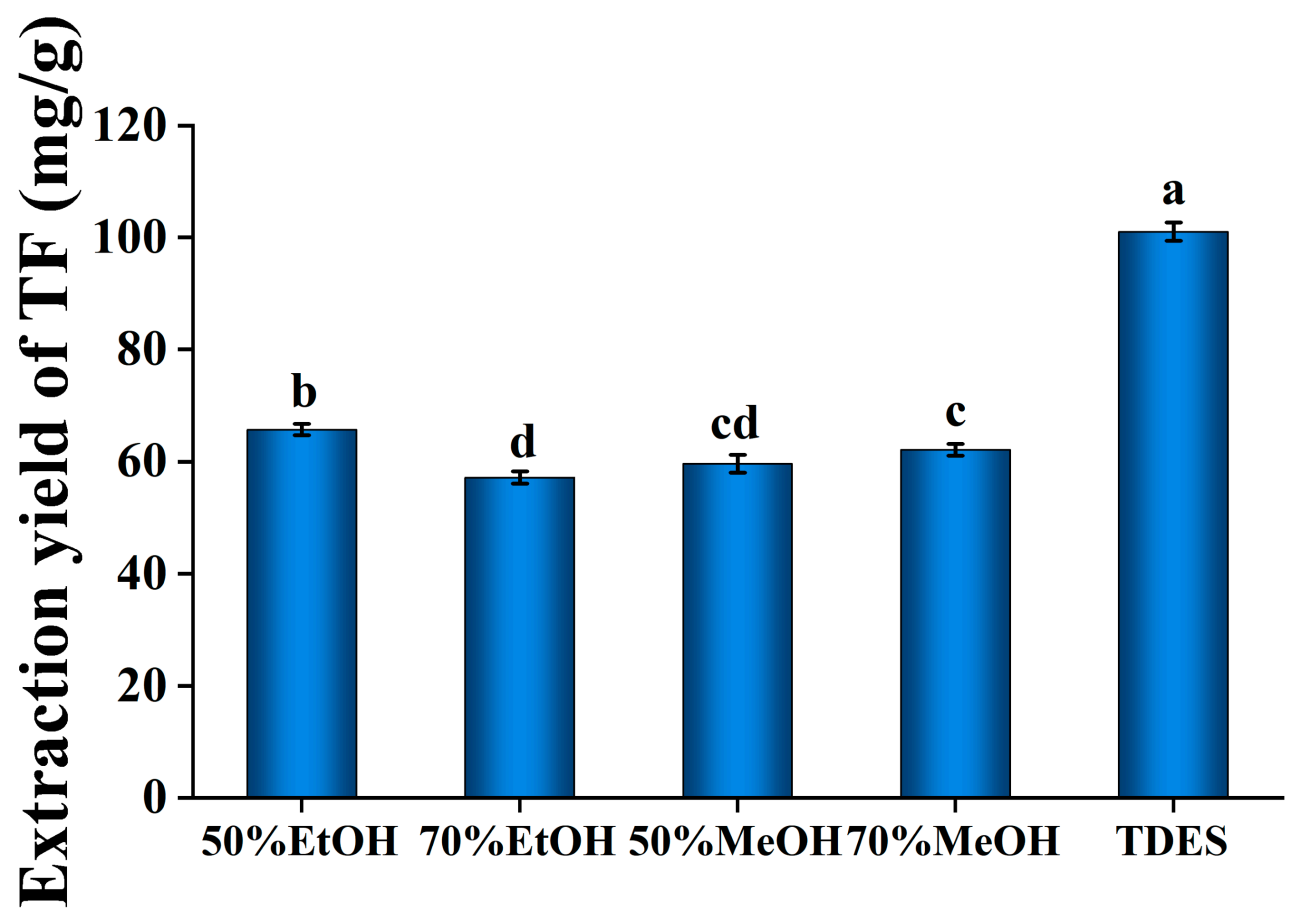
Comparison of TF extraction yield by traditional solvents. Different lowercase letters above bars indicate significant differences (*p* < 0.05) according to Tukey’s HSD test.

The TDES system (Gly-LevA-Xly) was compared with the traditional single-phase extraction system (50% methanol, 70% methanol, 50% ethanol, 70% ethanol) under the same conditions for extraction. The TF content extracted under the same process conditions is shown in Figure 7. The TF content extracted by the TDES system is higher than that of the traditional single-phase extraction system (1.50–1.77 times). Compared with the traditional single-phase extraction system, the TDES system developed in this study has more efficient extraction of TF and is environmentally friendly.

### 3.8. SEM of Residues Obtained by Different Extraction Methods

**Figure 8:**
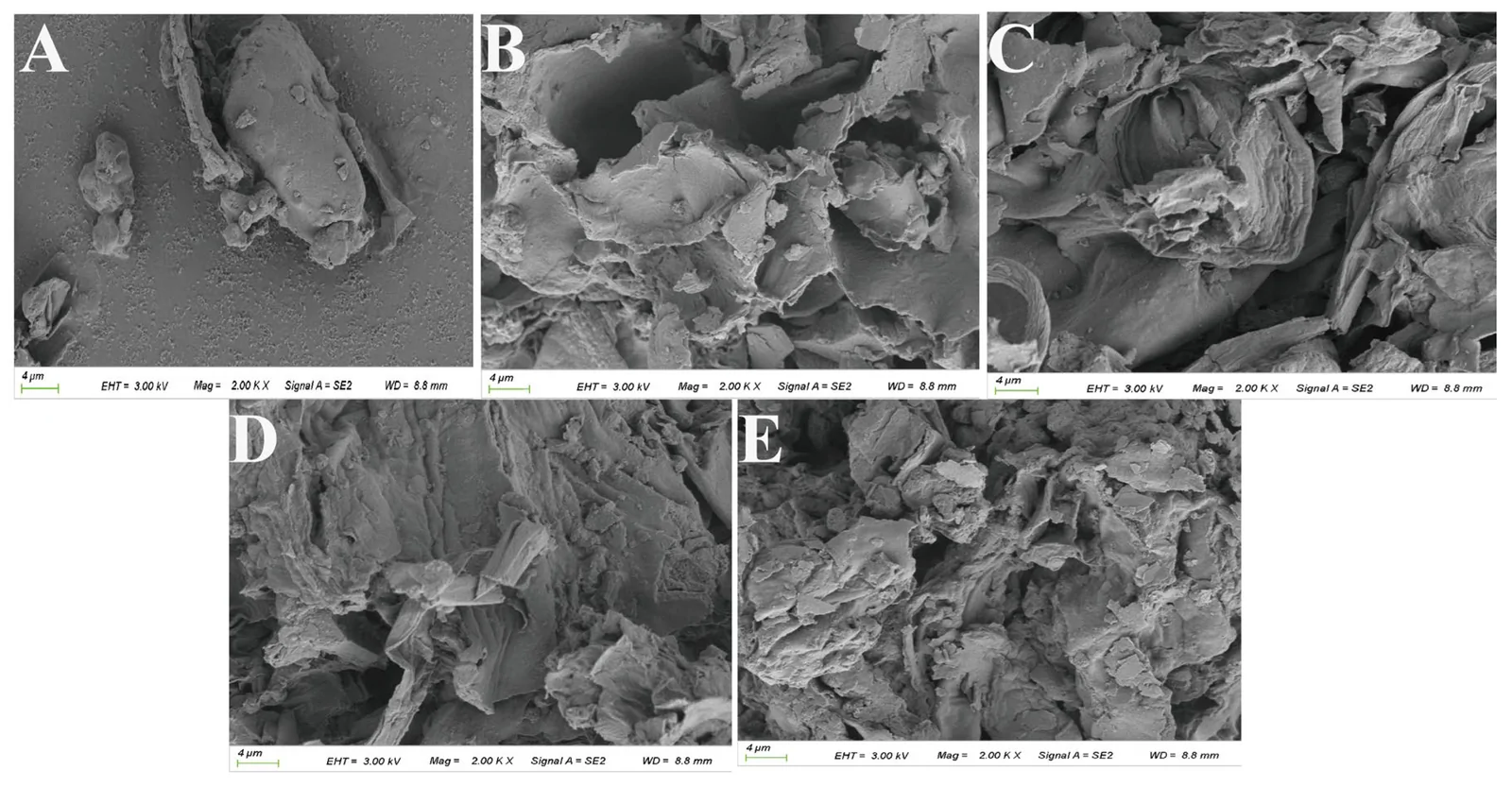
SEM images of *A. argyi* powder under different extraction treatments ((**A**) Raw material; (**B**) TDES-UAE; (**C**) 50% EtOH-UAE; (**D**) ME; (**E**) PE).

The microstructural changes of *A. argyi* powder under different extraction methods were examined by SEM (Figure 8). The untreated powder exhibited intact, smooth surfaces with preserved cell wall integrity (A). TDES-ultrasonic extraction caused severe fragmentation, forming a porous, sponge-like structure with near-complete cell wall disruption (B). In contrast, 50% ethanol extraction produced moderate wrinkling and local collapse (C), while TDES-impregnation (D) and TDES-percolation (E) showed only slight surface erosion. The damage severity followed the order: B > C > E > D > A. The fragmentation effect of ultrasonic-assisted TDES is better than that of other extraction methods; the ultrasonic cavitation effect can efficiently damage the cell wall, while the strong hydrogen bond effect of TDES accelerates the release of intracellular substances, thereby improving the extraction efficiency [58].

### 3.9. TDES Recycling Performance and TF Recovery Results

**Figure 9:**
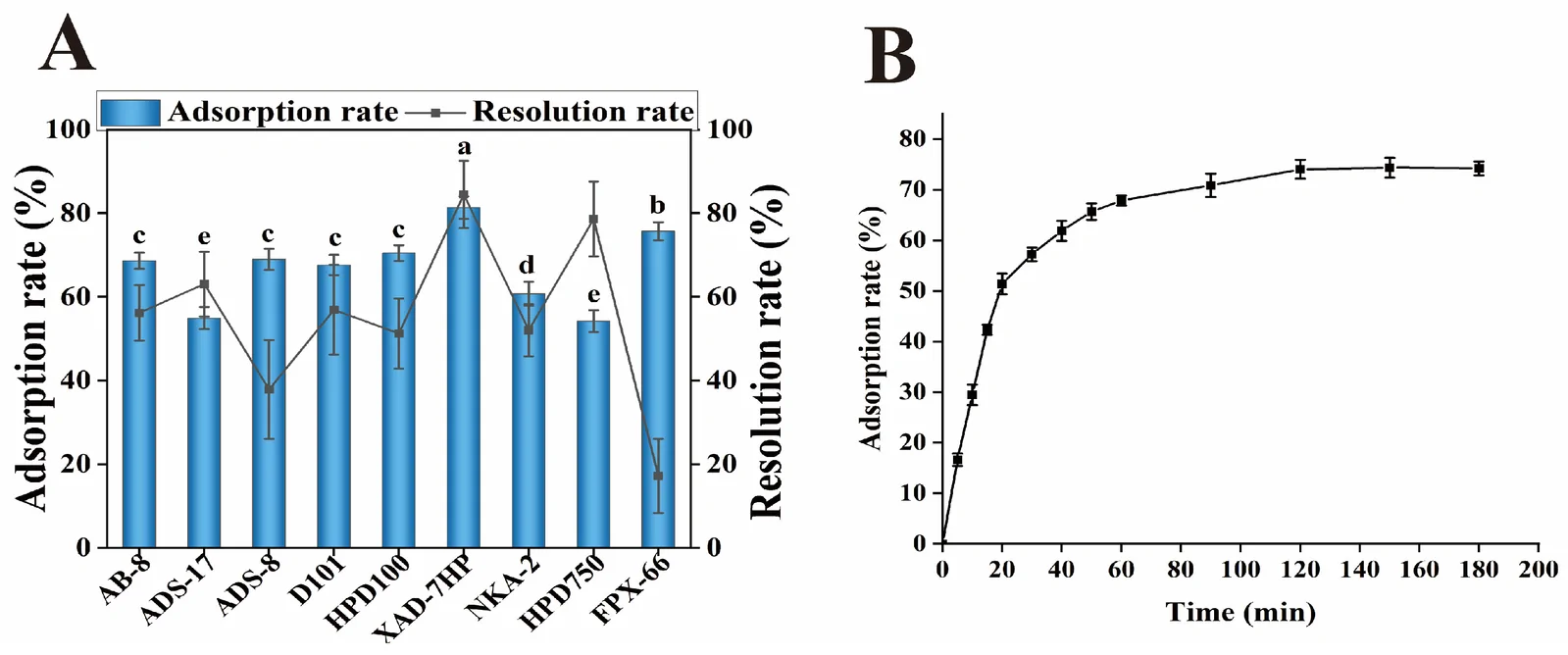
(**A**) Adsorption and desorption effects of macroporous resins on TF; (**B**) Static adsorption curves of XAD-7HP macroporous resin. Different lowercase letters above bars indicate significant differences (*p* < 0.05) according to Tukey’s HSD test.

The purification and solvent recycling performance of the TDES system was systematically evaluated. Nine macroporous resins were screened for TF enrichment, with XAD-7HP exhibiting the highest adsorption (81.3%) and desorption (70.3%) efficiencies (Figure 9A). Static adsorption kinetic experiments showed that the adsorption process quickly reached equilibrium within 2 h, with the adsorption efficiency up to 74.0% (Figure 9B). In the elution condition optimization test, 70% ethanol was screened as the ideal eluent, yielding the highest total flavonoid recovery of 70 ± 1.6%, which was obviously superior to methanol and other ethanol concentration groups (Table S7).

The reusability of the prepared TDES was evaluated by conducting repeated extraction experiments with the recycled solvent. The results showed that the TDES maintained stable extraction performance in the first three cycles. The corresponding flavonoid recovery rates were 96.8%, 85.1% and 88.2%, while the relative extraction yields were 100%, 88.4% and 84.5%, respectively. By contrast, a noticeable performance drop occurred in the fourth cycle, with the recovery rate falling to 66.7% and the relative extraction yield decreasing to 78.0%. This decline was probably caused by the destruction of the internal hydrogen bond structure of DES and the continuous accumulation of impurities (Table S8). The decline in extraction efficiency during the 4th recycle cycle can be attributed to two synergistic factors. First, repeated extraction led to gradual accumulation of plant-derived impurities (e.g., polysaccharides, pigments) in the TDES system. These impurities occupy hydrogen bond sites on glycerol, levulinic acid, and xylitol, disrupting the stable hydrogen bond network required for efficient flavonoid solubilization, as evidenced by the shortened average hydrogen bond lifetime in recycled TDES observed in our preliminary MD tests [59]. Second, mild heating (60 °C) and ultrasonic cavitation during repeated extraction may cause partial degradation of TDES components, particularly the ester groups in levulinic acid, further weakening intermolecular interactions. These findings align with previous reports on DES recycling for plant extraction [60], suggesting that simple filtration through neutral alumina prior to reuse could mitigate impurity accumulation and extend TDES service life in future scaled-up applications.

Overall, the above results verified that the combination of macroporous resin purification and recycled DES is feasible and effective for sustainable separation and enrichment of TF. This technical route is also well consistent with the application characteristics of existing DES-based separation and purification methods [5].

### 3.10. Molecular Dynamics (MD) Simulation

Herein, molecular dynamics simulation was applied to reveal the microscopic mechanism responsible for the efficient extraction of TF from *A. argyi* by TDES at the atomic scale. Eupatilin, the major flavonoid ingredient in *A. argyi*, was chosen as the representative compound to explore the underlying extraction mechanism. Simulation outcomes revealed that the TDES (Gly-LevA-Xyl) could break the intrinsic interaction between plant cell wall components and eupatilin more efficiently than conventional 50% (*v*/*v*) aqueous ethanol. To further support this finding, we quantitatively compared the hydrogen bonding behavior between different solvent systems and the plant matrix.

**Figure 10:**
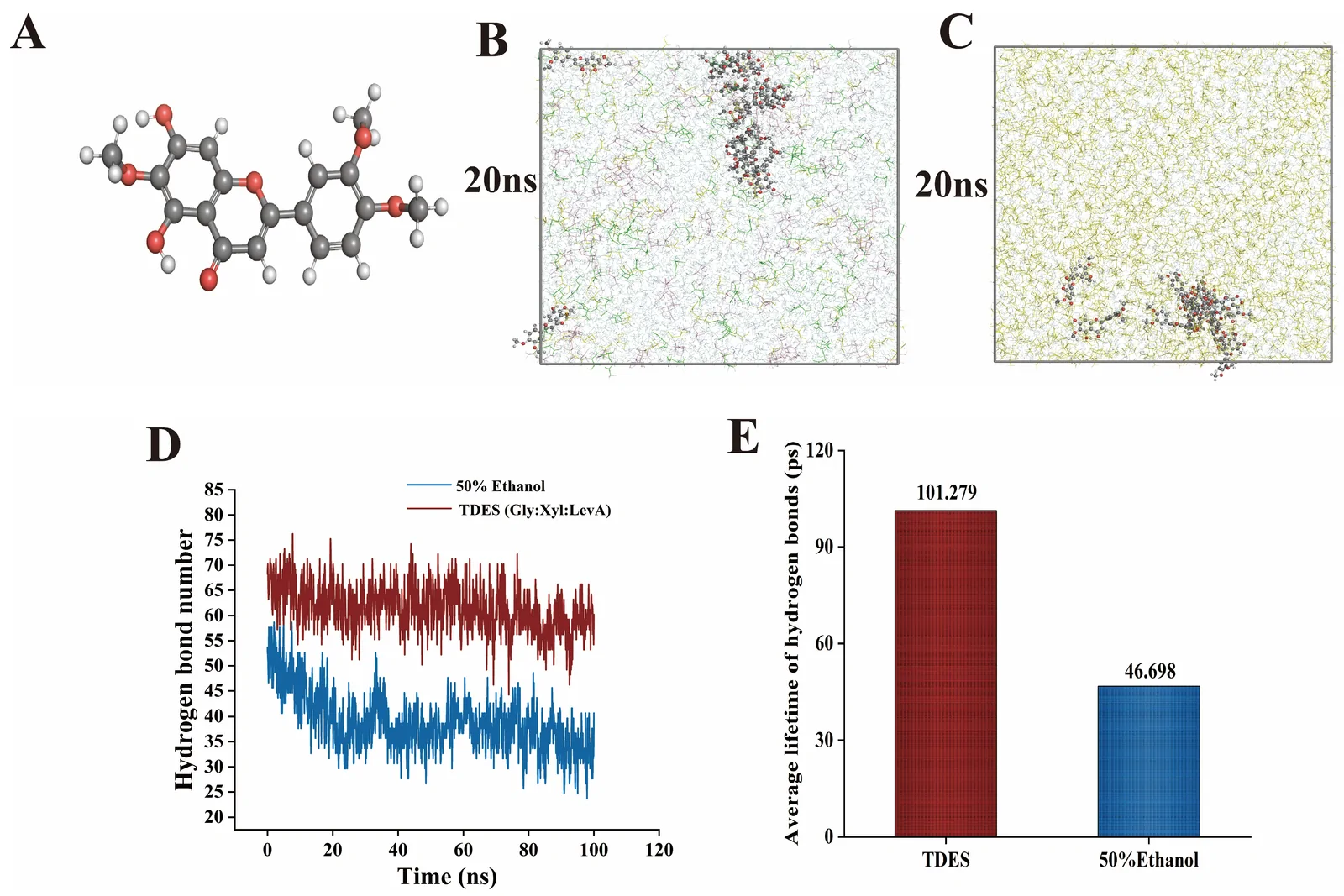
(**A**): Eupatilin structural formula; (**B**): Snapshots of Eupatilin dissolved in TDES; (**C**): Snapshots of Eupatilin dissolved in 50% ethanol; (**D**): Hydrogen bond number; (**E**): Average lifetime of hydrogen bonds.

As displayed in Figure 10B,C, eupatilin presented a more homogeneous dispersion state in the TDES system throughout the 20 ns simulation period relative to the 50% ethanol group. Figure 10D depicts the dynamic variation in hydrogen bond quantity for both solvent systems over simulation time. Though the two systems had similar total hydrogen bond numbers, the TDES group showed much milder fluctuation, reflecting more stable intermolecular interactions. As summarized in Figure 10E, the average hydrogen bond lifetime of the TDES–plant matrix system reached 101 ps, far higher than the value of 46.7 ps obtained for 50% ethanol. To further evaluate the binding stability of eupatilin within the TDES system, molecular dynamics (MD) simulations were conducted. As shown in Figure S4, the time profiles of RMSD, Rg, and SASA for both the complex and the ligand demonstrated that the system reached equilibrium and maintained high structural integrity throughout the 20 ns simulation, confirming the reliability of the predicted binding mode.

This finding agrees well with previous research conclusions. In TDES systems, multiple components are capable of constructing an intricate and dynamically stable hydrogen bond network. Such a stable network enables TDES to compete intensely with the original hydrogen bonds between cell wall polysaccharides (e.g., cellulose) and endogenous flavonoids, thereby destroying the binding state of target compounds in plant tissues and eventually realizing high-efficiency flavonoid extraction [61].

### 3.11. Quantum Chemical Insights into TDES-Cellulose Interactions

To elucidate the molecular origin of TDES’s superior extraction efficiency over conventional aqueous ethanol, density functional theory (DFT) calculations were performed using cellobiose—the β-1,4-linked disaccharide repeating unit of cellulose and the core structural component of *A. argyi* cell walls—as a model substrate. Geometry optimizations and frequency analyses were conducted at the B3LYP-D3(BJ)/def2-SVP level; single-point energies for high-precision binding evaluation were calculated at the def2-TZVP basis set level (see Section S1.7 for full computational details). Binding energies (ΔE) were defined via the supermolecular approach as ΔE = E(complex) − E(solvent) − E(cellobiose), with the SMD solvation model applied during single-point calculations to reflect the aqueous TDES environment (30% *w*/*w* water).

**Figure 11:**
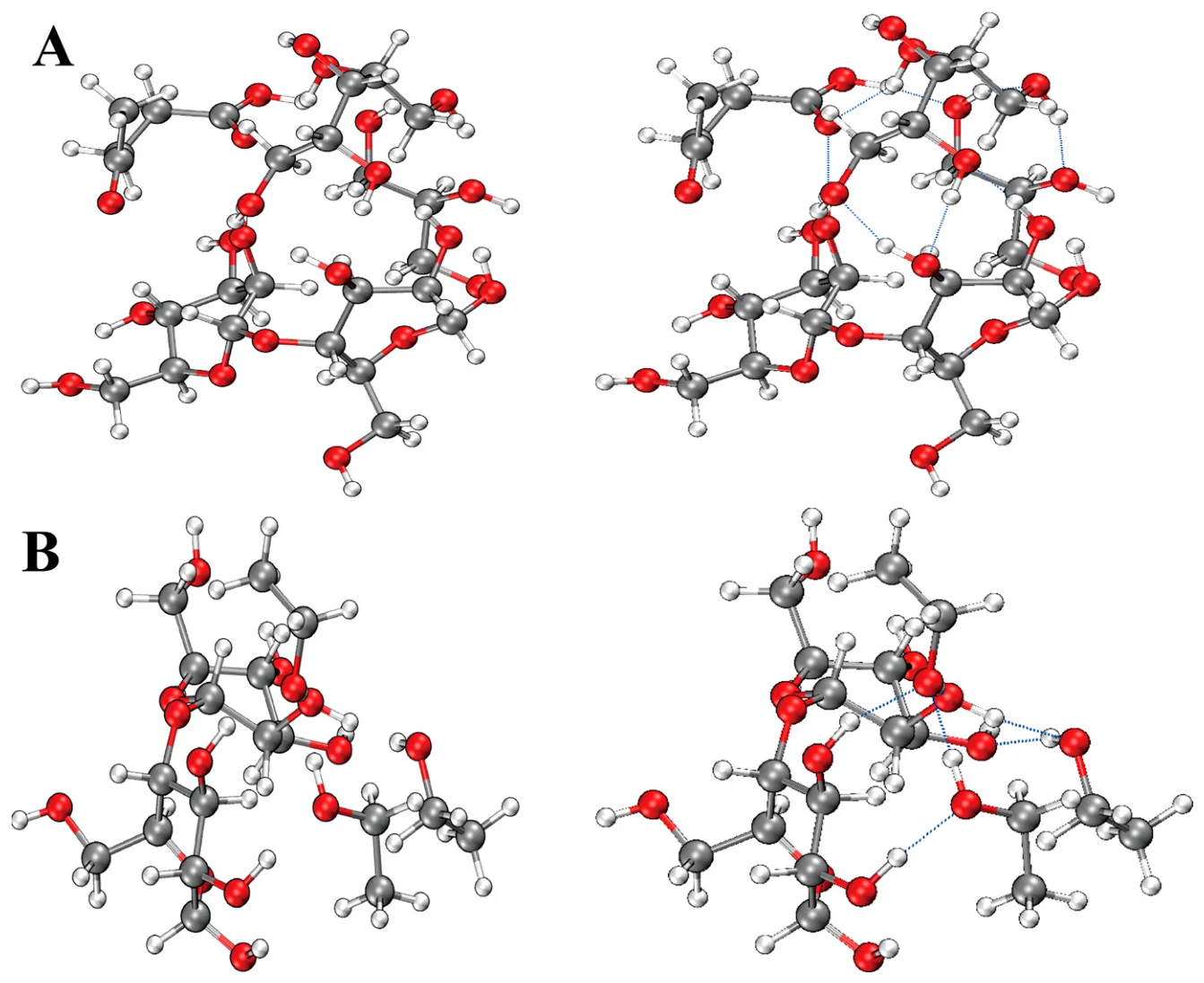
Comparative hydrogen bonding interaction topologies between cellobiose and different solvents. (**A**) TDES system (Gly/LevA/Xyl): The multi-component architecture facilitates a dense, multi-site hydrogen bonding network with cellobiose, forming 7–9 hydrogen bonds (blue dashed lines) anchored by diverse polar functional groups. (**B**) Ethanol system: Due to its monolithic structure and single hydroxyl group, ethanol forms only 1–2 hydrogen bonds with cellobiose via a limited, single-site interaction mode. Gray, red and white spheres represent carbon, oxygen and hydrogen atoms, respectively.

As summarized in Table S9, the gas-phase binding energy of the ethanol–cellobiose complex (−24.67 kcal/mol) was more negative (deeper in magnitude) than that of the TDES–cellobiose complex (−18.67 kcal/mol). This indicates that monolithic ethanol possesses deeper instantaneous single-point adsorption in vacuum. No claim of a superior absolute binding affinity for TDES based on gas-phase ΔE alone is made in this study. The mechanistic distinction lies entirely in interaction topology and network persistence. Constrained by its single hydroxyl group and compact structure, ethanol engages cellobiose through only 1–2 single-site hydrogen bonds (Figure 11B). In stark contrast, TDES leverages its multi-component architecture—Gly (H-bond donor/acceptor), LevA (carboxylic H-bond donor), and Xyl (polyol donor)—to establish a dense, multi-anchor hydrogen bond network with cellobiose, forming 7–9 hydrogen bonds anchored at diverse polar functional groups (Figure 11A). This “anchor-and-wrap” topology physically competes with and disrupts the intramolecular H-bond network responsible for cellulose crystallinity—a prerequisite for intracellular flavonoid release that single-site adsorption cannot achieve.

To assess the effect of the experimental medium, bulk aqueous solvation was incorporated via the SMD model (mimicking 30% water content). Absolute binding energies for both systems were attenuated by ~15% relative to vacuum; however, the multi-site H-bond geometry of TDES was structurally retained under solvated conditions, whereas ethanol’s single-anchor mode remained topologically insufficient to deconstruct cellulose packing. Crucially, gas-phase ΔE values and macroscopic solvent extraction efficiency should not be linearly extrapolated: the dynamic advantage of TDES is reflected in its H-bond persistence, not in a more negative single-point energy.

This DFT topological picture is dynamically corroborated by the MD simulations (Section 3.10), where TDES–cellobiose H-bonds survived 117% longer (average lifetime: 101.279 ps vs. 46.698 ps for 50% ethanol) with markedly milder bond-number fluctuations. The persistent solvation shell formed by TDES around cellulose chains prevents their re-aggregation post-disruption and sustains mass transfer of released flavonoids—directly explaining the severe cell-wall porosity observed in SEM (Section 3.8) and the 2.1-fold higher total flavonoid yield of TDES versus 50% aqueous ethanol (Section 3.4 and Section 3.7).

In summary, TDES outperforms aqueous ethanol in cell-wall deconstruction and flavonoid extraction not because of a more favorable gas-phase binding energy, but because of its multi-anchor H-bond topology (7–9 bonds), structural resilience in aqueous medium, and dynamically persistent interaction lifetime—a multi-scale mechanistic picture consistently supported by DFT topology, MD lifetime, SEM morphology, and experimental extraction yields.

### 3.12. In Vitro Antioxidant Activity

**Figure 12:**
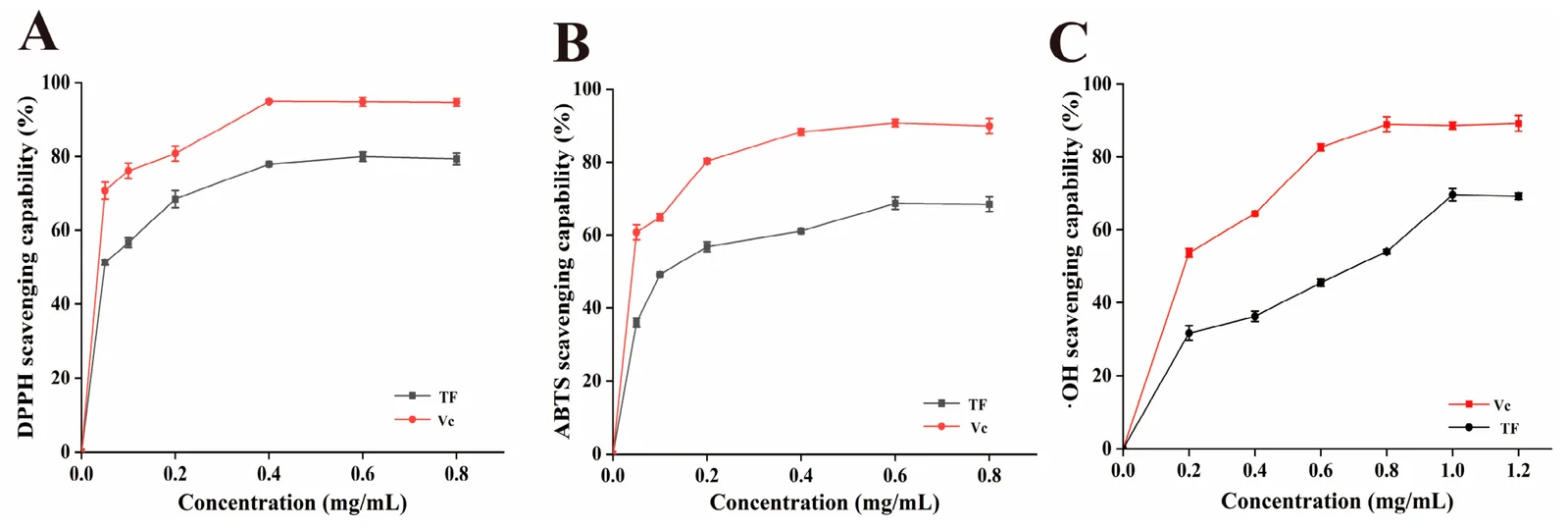
Free radical scavenging assay ((**A**) DPPH radical scavenging activity; (**B**) ABTS radical scavenging activity; (**C**) hydroxyl radical scavenging activity. DPPH solution (0.05–0.8 mg/mL), ABTS solution (0.05–0.80 mg/mL), and hydroxyl radical solution (0.20–1.20 mg/mL)).

In this study, we evaluated the in vitro antioxidant activity of TF from *A. argyi* via three widely used free radical scavenging methods: DPPH, ABTS, and hydroxyl radical (·OH) scavenging. Consistent with common experimental protocols for antioxidant evaluation, Vitamin C (Vc) was employed as a positive control to benchmark TF’s activity. As illustrated in Figure 12, both TF and Vc showed a distinct concentration-dependent increase in their ability to scavenge all three free radicals, within the concentration ranges tested 0–0.8 mg/mL for DPPH and ABTS assays, and 0–1.2 mg/mL for the ·OH scavenging assay.

For the DPPH radical scavenging assay (Figure 12A), we determined an IC_50_ value of 0.176 (0.152–0.183) mg/mL for TF. At the maximum tested concentration of 0.8 mg/mL, Vc achieved a scavenging rate of 94.9%, whereas TF reached 77.9%, indicating a lower but still considerable scavenging capacity for TF. In the ABTS radical scavenging test (Figure 12B), the IC_50_ of TF was 0.159 (0.148–0.170) mg/mL; at the highest concentration assayed, Vc and TF showed maximum scavenging rates of 90.8% and 68.7%, respectively. Regarding the ·OH scavenging assay (Figure 12C), TF had an IC_50_ of 0.767 (0.712–0.88) mg/mL. Even when the concentration was increased to 1.2 mg/mL (the highest level tested), Vc still outperformed TF, with scavenging rates of 88.9% and 69.6%, respectively.

These findings suggest that the antioxidant mechanism of TF is likely different from that of Vc, which primarily acts through a single, rapid hydrogen-donating pathway. As reported in previous studies, flavonoids typically exert their antioxidant effects through multiple pathways, such as hydrogen atom transfer, single electron transfer, and metal ion chelation, and they often show stronger scavenging activity against highly reactive radicals like ·OH. Taken together, the results confirm that TF from *A. argyi* has broad-spectrum and considerable free radical scavenging activity, with notable potential in scavenging ·OH. This makes it a valuable natural antioxidant resource with good development prospects. This broad-spectrum scavenging capacity aligns with recent spectroscopic evidence of flavonoid-mediated free radical quenching via electron transfer and hydrogen donation pathways [62].

### 3.13. Molecular Interaction of Eupatilin with DPPH and ABTS Radicals: A Structural-Level Hypothesis

To elucidate the molecular mechanism behind how eupatilin interacts with free radical scavengers (DPPH and ABTS), we used molecular docking technology to systematically analyze the binding modes and interaction features of the ligand–receptor complexes formed between them. For the conformational analysis and interaction interpretation of these docking complexes, we employed Discovery Studio Visualizer 2024; meanwhile, PyMOL 3.0 was used to visualize their three-dimensional structures. The molecular docking results showed that eupatilin—the key bioactive component of TF from *A. argyi* can bind stably to both DPPH and ABTS radicals.

**Figure 13:**
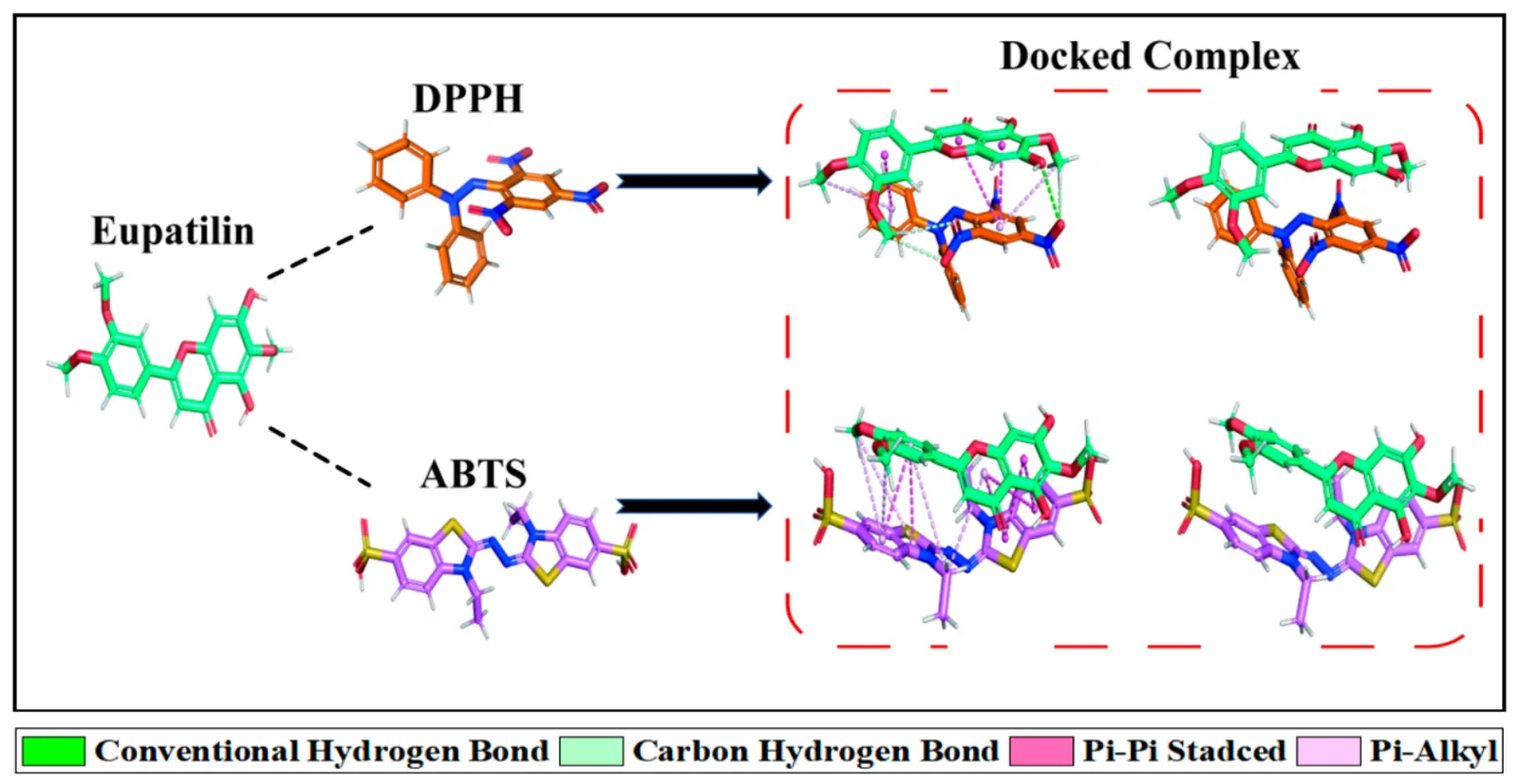
Molecular docking of Eupatilin with DPPH and ABTS radicals.

As detailed in Table S10 and Figure 13, the binding energy of eupatilin with ABTS was −4.515 kcal/mol, while that with DPPH was −4.083 kcal/mol. According to the commonly used evaluation standards for molecular docking binding energies, a value lower than −7 kcal/mol means strong binding, and a range of −4 to −7 kcal/mol indicates moderate binding. Based on this, our results confirm that eupatilin has a moderate binding affinity for both radicals. These docking results illustrate plausible flavonoid–radical interaction modes; however, the experimental IC_50_ values (DPPH: 0.176 mg/mL; ABTS: 0.159 mg/mL) were determined for the total flavonoid fraction, not for isolated eupatilin. Therefore, the docking serves as an in silico structural hypothesis for the TF mixture’s radical-quenching profile rather than a mechanistic proof for a single purified compound.

A more in-depth analysis of their interaction patterns revealed clear differences in the binding mechanisms between the two complexes. When eupatilin binds to DPPH, the interaction involves one hydrogen bond, three carbon–hydrogen bonds, and multiple hydrophobic interactions, specifically three π–π stacking interactions and three π–alkyl interactions. The presence of hydrogen bonds here aligns with the classic mechanism of free radical quenching, where hydrogen is directly donated to neutralize DPPH radicals.

In contrast, the binding of eupatilin to ABTS^+^ is mainly driven by hydrophobic interactions, which include six π–π stacking interactions and five π–alkyl interactions, with no hydrogen bonds formed at all. This interaction pattern is consistent with the theoretical model for neutralizing cationic radicals, which relies on π-electron delocalization or single-electron transfer (SET). These different interaction modes are a reflection of the distinct chemical properties and reaction pathways of DPPH and ABTS radicals, and together they clarify the structural basis for the multi-mechanism antioxidant activity of eupatilin [63]. These computational findings are strongly supported by our experimental antioxidant results: the moderate binding energies (−4.083 to −4.515 kcal/mol) correspond well with the moderate IC_50_ values of TF against DPPH (0.176 mg/mL) and ABTS (0.159 mg/mL), as binding affinity directly correlates with radical scavenging efficiency [24]. Furthermore, recent studies confirm that flavonoid-radical interactions mediated by hydrogen bonding and π-π stacking are the dominant mechanisms for DPPH and ABTS quenching in vitro [64], validating our docking-derived interaction models. Notably, the lack of hydrogen bonds between eupatilin and ABTS^+^ aligns with the cationic nature of ABTS radicals, which preferentially interact via hydrophobic π-π stacking rather than polar hydrogen bonding, a feature consistently reported in recent flavonoid docking studies [65].

### 3.14. Protective Effect of TF Against Oxidative Damage to Cells

**Figure 14:**
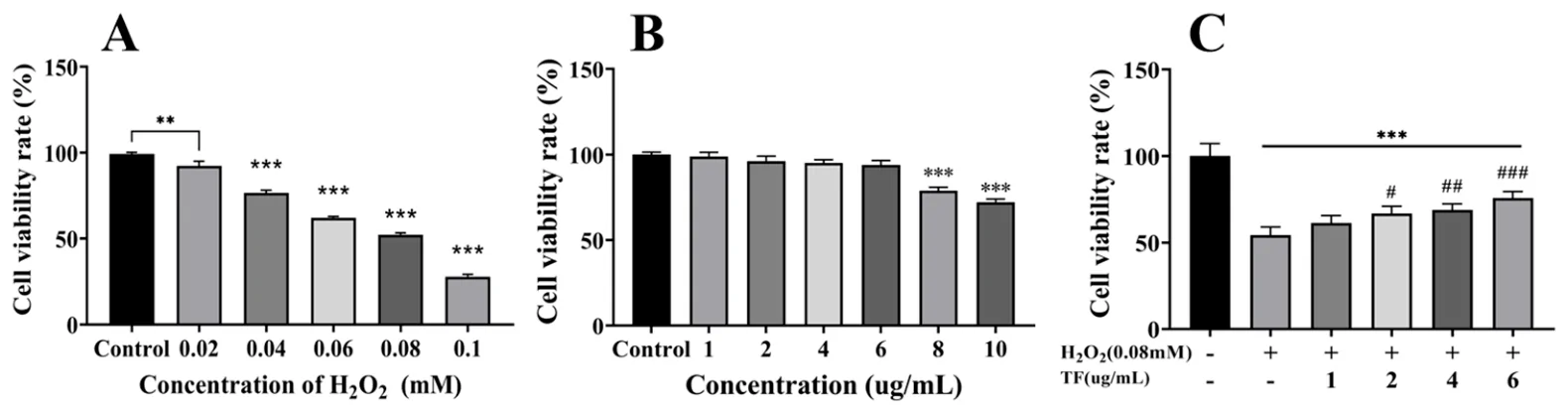
Protective effect of TF against oxidative damage to cells. (**A**) Effect of different concentrations of H_2_O_2_ on HepG2 cells; (**B**) Effect of different concentrations of TF on HepG2 cells; (**C**) Protection of TF against H_2_O_2_-induced oxidative damage in a model of HepG2 cells. Data were expressed as mean ± S.D. (*n* = 3), ** *p* < 0.01, *** *p* < 0.001 compared to control group; # *p* < 0.05, ## *p* < 0.01, ### *p* < 0.001 compared to H_2_O_2_ model group.

Within 4 h, the damage that H_2_O_2_ caused to HepG2 cells was concentration-dependent (Figure 14A). At 0.08 mmol/L H_2_O_2_, cell viability dropped to 52.7%, a result that was significantly different from untreated cells (*p* < 0.001). We picked this 0.08 mmol/L dose for later modeling, since it hit the right balance: it induced enough oxidative damage but still kept enough cells alive.

We first checked the cytotoxicity of TF itself to make sure our assessment of its protective effect was reliable. As Figure 14B shows, HepG2 cells treated with 1–6 μg/mL TF for 24 h had almost the same viability as untreated cells (>96%, *p* > 0.05). But at higher concentrations (8–10 μg/mL), cell viability dropped significantly, meaning TF caused cytotoxic damage here. This safety check confirmed TF did not have obvious cytotoxicity at 1–6 μg/mL. So any protective effects we saw in this range came from its real antioxidant activity, not cytotoxic side effects [66].

TF showed significant protection against H_2_O_2_-induced cell damage. H_2_O_2_ treatment reduced cell viability to 54.2% (*p* < 0.001), successfully establishing an oxidative damage model (Figure 14C). TF pretreatment at 4 and 6 μg/mL significantly protected cells, with viability reaching 75.7%. In contrast, 1–2 μg/mL TF had little protective effect, indicating a threshold concentration is needed for TF to activate its antioxidant defense. At lower concentrations, TF’s antioxidant capacity is insufficient to neutralize H_2_O_2_-generated reactive oxygen species; higher concentrations elicit a concentration-dependent protective response.

The concentration-dependent protective effect of TF likely stems from its ability to scavenge reactive oxygen species (ROS) and modulate cellular antioxidant defense pathways. Previous studies report that *A. argyi* flavonoids activate the Nrf2/ARE signaling pathway, upregulating endogenous antioxidant enzymes (e.g., SOD, CAT) to reduce oxidative stress [67]. Additionally, eupatilin, the dominant flavonoid in our extract, has been shown to suppress mitochondrial apoptosis by downregulating the Bax/Bcl-2 ratio and inhibiting caspase-3 activation in H_2_O_2_-injured cells [68]. While this study did not quantify ROS levels or apoptosis markers, future work will integrate multi-omics approaches to elucidate the precise molecular mechanisms underlying TF-mediated cytoprotection.

### 3.15. In Vitro Anti-Tumor Activity Results

**Figure 15:**
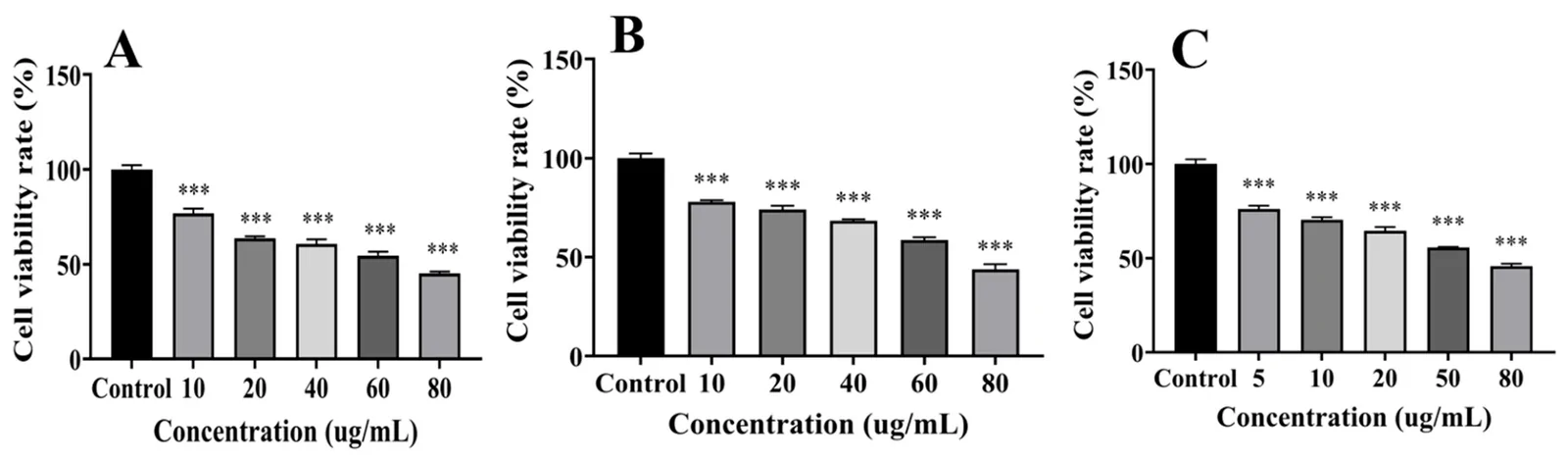
Anti-tumor activities of TF. ((**A**) is HCT116; (**B**) is HepG2; (**C**) is A549). Data were expressed as mean ± S.D. (*n* = 3), *** *p* < 0.001 compared to control group.

To assess the antitumor potential of TF from *A. argyi*, we used the CCK-8 assay to test its effects on the proliferation of three cancer cell lines: colorectal cancer (HCT116), hepatocellular carcinoma (HepG2), and lung cancer (A549). As Figure 15 shows, TF inhibited the growth of all three cell lines in a clear concentration-dependent manner. The calculated IC_50_ values were quite similar: 50.5 (95% CI: 44.3–56.7) μg/mL for HCT116, 49.7 (95% CI: 43.5–55.9) μg/mL for A549, and 49.8 (95% CI: 43.8–55.8) μg/mL for HepG2 cells. At the highest tested concentration (80 μg/mL), TF’s proliferation inhibition on all cell lines was extremely significant (*p* < 0.001).

This study examined TF’s antiproliferative effects on the three aforementioned human cancer cell lines. A key finding was that TF had similar inhibitory efficacy across all three cell types, with IC_50_ values around 50 μg/mL, suggesting its antitumor mechanism may be broad-spectrum rather than specific to one cell lineage. This broad-spectrum activity likely comes from the multi-targeting nature of flavonoids. Accumulating evidence indicates that the antitumor effects of *A. argyi* extracts arise from the cooperative actions of multiple flavonoids rather than a single constituent—for instance, eupatilin suppresses malignancy in pancreatic cancer cells, while jaceosidin and hispidulin inhibit proliferation in cervical and colorectal cancer cells, respectively. As reported, these compounds inhibit cancer cell proliferation via multiple pathways: inducing cell cycle arrest, activating mitochondrial apoptosis, and suppressing pro-survival signaling like Akt/ERK. The comparable IC_50_ values (~50 μg/mL) across three cancer cell lines observed in this study likely reflect the integrated antitumor effect of the TF fraction, wherein eupatilin and other co-existing flavonoids act in concert. The similar IC_50_ values provide preliminary experimental evidence that TF could be a potential broad-spectrum plant-derived antitumor candidate.

### 3.16. Study on Enzyme Inhibition Activity In Vitro

**Figure 16:**
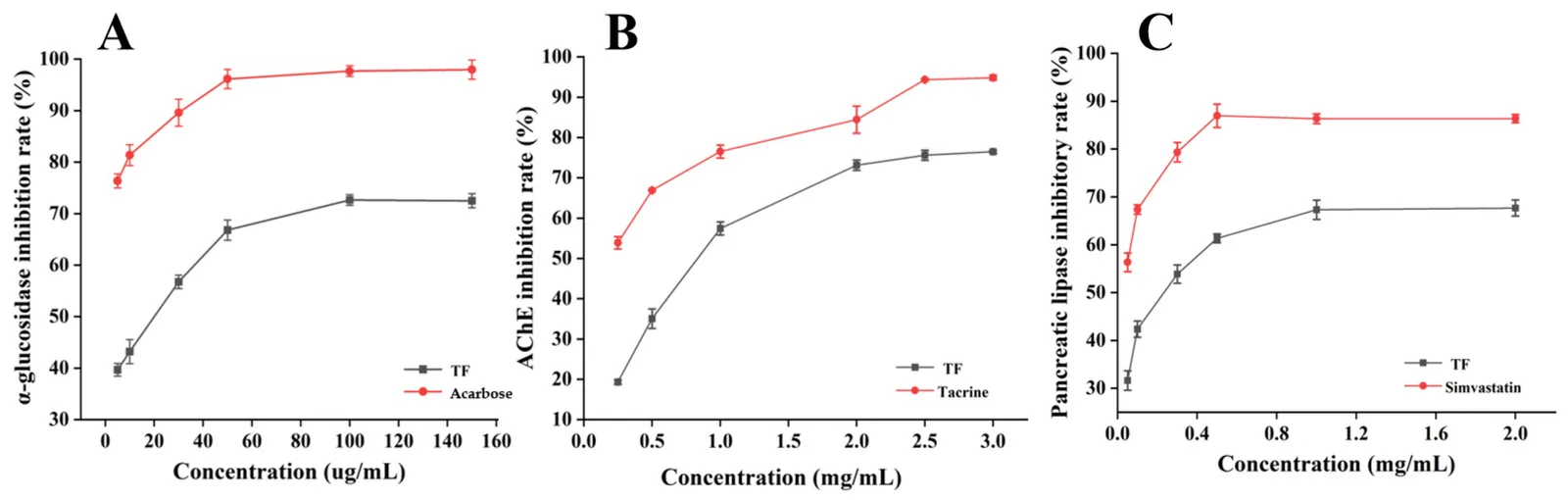
Enzyme inhibitory activity ((**A**) α-glucosidase inhibitory activity; (**B**) acetylcholinesterase inhibitory activity; (**C**) porcine pancreatic lipase inhibitory activity).

We evaluated the multi-target biological activity of TF from *A. argyi* using in vitro enzyme inhibition assays, with results shown in Figure 16. TF inhibited α-glucosidase, acetylcholinesterase (AChE), and porcine pancreatic lipase in a clear concentration-dependent manner. This multipotent regulatory profile is consistent with the emerging trend that plant-derived flavonoid fractions act as multi-target agents for metabolic syndrome management, wherein the combined effects of structurally diverse flavonoids contribute to the integrated bioactivity. At 100 μg/mL, TF’s inhibition rate against α-glucosidase was 72.6%, with an IC_50_ of 55.3 (51.2–59.4) μg/mL. Though this IC_50_ was higher than positive control acarbose, it confirms TF’s potential for glycemic regulation. At 2.5 mg/mL, TF inhibited AChE by 75.6% (0.618 (0.572–0.664) mg/mL), suggesting neuroprotective effects possibly via modulating the cholinergic system to improve cognitive function. Additionally, at 1.2 mg/mL, TF’s inhibition rate against porcine pancreatic lipase was 67.3% (0.125 (0.116–0.134) mg/mL), indicating potential for regulating lipid metabolism and lowering blood lipids. These results show TF from *A. argyi* has promising prospects for developing adjunctive therapeutics or functional foods for diabetes, Alzheimer’s disease, and obesity-related metabolic syndrome. This multipotent regulatory effect is consistent with emerging trends in natural product research, where plant-derived flavonoids serve as multi-target agents for metabolic syndrome management [67].

### 3.17. Molecular Docking Interaction Analysis of Eupatilin—Xanthine Oxidase Complex

As an in silico exploration, eupatilin—the most abundant quantified flavonoid in the optimized TDES extract—was docked into the XO active site (PDB 1FIQ) to probe potential binding modes. XO is a key enzyme in uric acid biosynthesis and a major source of reactive oxygen species—molecular docking studies were performed using the XO crystal structure (PDB ID: 1FIQ) [44]. The co-crystal ligand position was used as the docking site. As summarized in Table S11, eupatilin exhibited a binding energy of −7.778 kcal/mol, which was lower (i.e., more favorable) than that of the clinical XO inhibitor allopurinol (−6.86 kcal/mol), indicating that eupatilin possesses a stronger predicted binding affinity for the XO active pocket.

**Figure 17:**
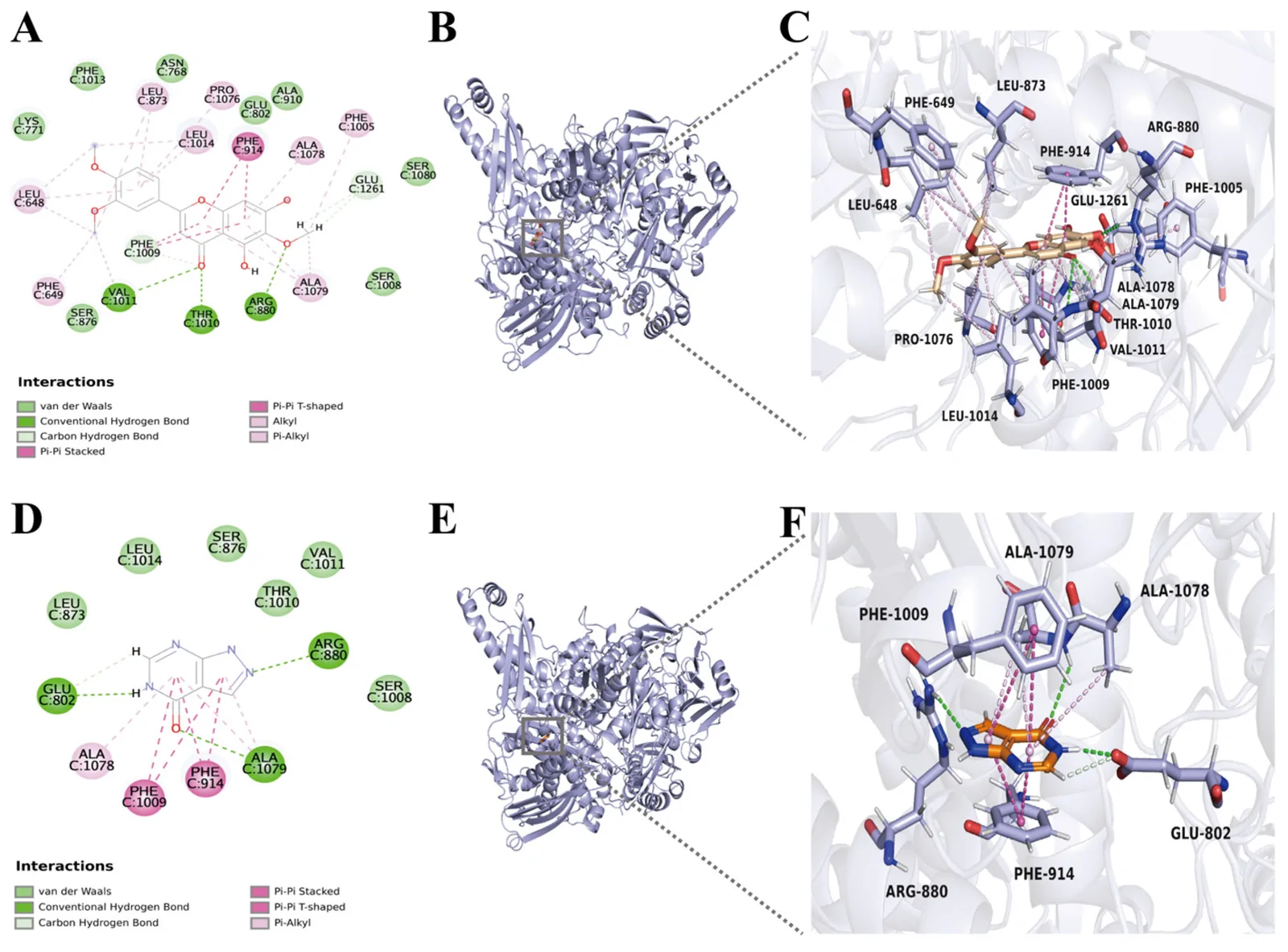
Molecular docking analysis of eupatilin with xanthine oxidase (XO). (**A**) 2D interaction network of eupatilin within the XO active site, highlighting hydrogen bonds (green dashed lines), hydrophobic interactions (gray lines), and π-π stacking (purple lines). Key interacting residues are labeled. (**B**) Overall 3D binding conformation of eupatilin (orange sticks) bound to the XO protein (blue ribbon). The catalytic pocket and secondary structure elements are shown. (**C**) Close-up view of the binding pocket detailing the spatial arrangement between eupatilin and key residues (e.g., GLU-802, ALA-1079, ARG-880). (**D**) 2D interaction map of the reference ligand allopurinol, illustrating its binding pattern with surrounding residues. (**E**) 3D conformation of the allopurinol-XO complex. (**F**) Detailed interactions of allopurinol with active site residues, emphasizing the hydrogen bonding network.

Detailed interaction patterns are illustrated in Figure 17 and Table S11. Eupatilin formed three hydrogen bonds with Thr1010, Val1011, and Arg880, and three carbon–hydrogen bonds with Phe1009 and Glu1261. In addition, extensive hydrophobic interactions were observed, including π–π stacking (Phe914), π–π T-shaped (Phe1009), alkyl (Leu873, Leu648, Leu1014, Ala1079, Val1011), and π–alkyl (Pro1076, Phe1005, Ala1078, Leu648, Leu873, Leu1014, Ala1079, Phe649) contacts. In comparison, allopurinol formed three hydrogen bonds (Glu802, Ala1079, Arg880) and one carbon–hydrogen bond (Glu802), along with π–π stacking (Phe914) and π–π T-shaped (Phe1009) interactions, but fewer hydrophobic contacts overall.

These in silico results suggest that eupatilin can stably occupy the XO active pocket through a combination of polar and non-polar interactions, providing a structural basis for its potential XO-inhibitory and indirect antioxidant activity. This finding complements the multi-target enzyme inhibition profile of the TF fraction (Section 3.15) and highlights eupatilin as a promising natural candidate for the development of XO inhibitors [33]. It should be noted that while the present study did not perform an independent in vitro XO inhibition assay using eupatilin purified from our *A. argyi* TDES extract, the XO inhibitory activity of eupatilin has been experimentally demonstrated by Xu et al. [69]. In their comprehensive in vitro and in vivo study, eupatilin was shown to exert a clear inhibitory effect on XOD activity in vitro, and to significantly reduce serum UA, creatinine, BUN, IL-1β, and IL-6 levels in hyperuricemic rats, while inhibiting ADA and XOD enzyme activities in the liver and serum and modulating GLUT9, URAT1, and ABCG2 protein expression in the kidneys and ileum. The molecular docking results presented herein are therefore consistent with, and externally supported by, the published experimental findings of Xu et al. [69]. Nevertheless, experimental validation using the specific eupatilin standard purified from our TDES extract remains a planned follow-up investigation.

## 4. Conclusions

A food-grade ternary deep eutectic solvent (TDES, glycerol/levulinic acid/xylitol = 1:1:1, 30% *w*/*w* water) combined with ultrasound assistance enabled efficient, sustainable recovery of total flavonoids (TF) from *Artemisia argyi*, with a GA-ANN-optimized yield of 108 ± 1.7 mg/g—1.5–1.8-fold higher than conventional hydroalcoholic extraction. The method scored 0.70 (AGREEprep) and 80/100 (MoGAPI), confirming strong alignment with green analytical chemistry principles. Atomic- and micro-scale mechanistic investigations revealed that TDES disrupts plant cell walls via a stabilized, multi-site hydrogen bond network: MD simulations showed an average H-bond lifetime of 101.279 ps (vs. 46.698 ps for 50% ethanol), and DFT calculations at the B3LYP-D3(BJ)/def2-TZVP level confirmed that TDES anchors to cellobiose with 7–9 hydrogen bonds, far exceeding ethanol’s 1–2 single-site interactions—a finding that directly explains the severe wall porosity observed in SEM and the superior macroscopic extraction yield. Biologically, the purified TF fraction exhibited broad, multi-target activity: potent radical scavenging (DPPH IC_50_: 0.176 mg/mL; ABTS IC_50_: 0.159 mg/mL), enzyme inhibition relevant to metabolic syndrome (α-glucosidase IC_50_: 55.31 μg/mL; pancreatic lipase IC_50_: 0.125 mg/mL; AChE IC_50_: 0.618 mg/mL), and broad-spectrum antiproliferative effects against HCT116, A549, and HepG2 (IC_50_ ≈ 50 μg/mL). In cellular models, TF pretreatment (4–6 μg/mL) significantly protected HepG2 cells against 0.06 mmol/L H_2_O_2_-induced oxidative damage. Given its high abundance in the TDES extract (3.64 ± 0.05 mg/g), eupatilin was selected as a representative ligand for in silico docking against xanthine oxidase (XO, PDB 1FIQ), yielding a predicted binding affinity of −7.778 kcal/mol—more favorable than allopurinol (−6.86 kcal/mol). This docking exercise provides a structural-level illustration of potential flavonoid–XO interactions but does not imply that eupatilin is solely or primarily responsible for the biological effects observed for the total flavonoid mixture.

From a translational perspective, the practical value of this platform lies in its convergence of process scalability, product applicability, and circular bioeconomy. All TDES components are food-grade and inexpensive, and the solvent retained >84% extraction efficiency over three reuse cycles, which substantially lowers operational costs and supports the feasibility of industrial scale-up. The resulting TF extract is well positioned as a natural antioxidant alternative to synthetic additives such as TBHQ in baked goods, edible oils, and cosmetic emulsions; concurrently, its multi-enzyme inhibition profile (α-glucosidase, pancreatic lipase, AChE) underpins the development of nutraceuticals targeting glycemic control, weight management, and cognitive health. Importantly, this approach transforms *A. argyi* leaf biomass—often an underutilized agricultural byproduct—into high-value functional ingredients, thereby aligning with sustainable agriculture and circular bioeconomy strategies.

Limitations of the current work include the evaluation of total flavonoid mixtures rather than individually purified flavonoid standards across the bioactivity assays, and the lack of in vivo pharmacokinetic validation; both are being addressed in ongoing follow-up studies. Overall, this study delivers a robust, green, and mechanism-informed extraction platform and positions *A. argyi* TF as a multipotent natural candidate for functional food and nutraceutical sectors.

## Data Availability

Data will be made available on request.

## Supplementary Materials

The following supporting information can be downloaded at: https://www.mdpi.com/article/10.3390/antiox15091069/s1, Table S1: The calibration curves, linear ranges, LODs and LOQs; Table S2: Results of recovery tests for the four flavonoids (*n* = 9); Table S3: List of prepared TDESs; Table S4: Box-Behnken design table of factor levels; Table S5: Box-Behnken Experimental Results; Table S6: Analysis of Variance for the Regression Equation; Table S7: Investigation of elution concentration for methanol and ethanol; Table S8: Recovery of TDES; Table S9: Calculated single-point energies and binding energies of solvent–cellobiose complexes at the DFT level; Table S10: Molecular docking results; Table S11: Binding energies of eupatilin and allopurinol to xanthine oxidase; Figure S1: HPLC chromatograms of TF sample and mixed standard (1: Naringin, 2: Luteolin, 3: Kaempferol, 4: Eupatilin); Figure S2: Regression plots for training, validation, test, and all data Sets; Figure S3: (A) Regression analysis of TF yield prediction for training, validation, and test sets; (B) Error histogram of prediction errors for all datasets; (C) MSE convergence curve of the ANN during training; (D) GA fitness evolution curve over generations; Figure S4: Molecular dynamics simulation profiles over time. (A) RMSD curve of the TDES system over time; (B) Radius of gyration (Rg) curve of the TDES system over time; (C) Solvent accessible surface area (SASA) curve of the TDES system over time; (D) RMSD curve of eupatilin over time; (E) Radius of gyration (Rg) curve of eupatilin over time; (F) Solvent accessible surface area (SASA) curve of eupatilin over time.
