# Supplementary material for: Ultrasound-Assisted Ternary Deep Eutectic Solvent Extraction of Total Flavonoids from Artemisia argyi: GA-ANN Optimization, Greenness Assessment, and In Vitro Bioactivity Evaluation
Source: Antioxidants (Basel). 2026 Aug 26;15(9):1069. doi: 10.3390/antiox15091069 (PMC13603408; doi:10.3390/antiox15091069)
Supplement: Supplementary file 1 [file antioxidants-15-01069-s001.zip › antioxidants-4474101-supplementary.pdf]

Supplementary materials

Ultrasound-Assisted ternary deep eutectic solvent extraction of total flavonoids from *Artemisia argyi*: GA-ANN optimization, greenness assessment, and *in vitro* bioactivity evaluation

S1 Materials and methods

S1.1 Materials and reagents

Artemisia argyi (*A. argyi*) was purchased from Qichai Store, Qichun County, Hubei Province, China (voucher number: CRPV-20251217). The specimen was authenticated by Professor Yujin Zhang from the School of Pharmacy, Zunyi Medical University, Zunyi, China, as the dried leaves of *Artemisia argyi* Lev. et Vant. (Asteraceae). A voucher specimen has been deposited in the Laboratory 2-611, School of Pharmacy, Zunyi Medical University (accession number: ZMUH-2025-1217).

Quercetin (≥98%, HPLC grade), sodium nitrite (≥99%, analytical grade), aluminium nitrate nonahydrate (≥99%, analytical grade), sodium hydroxide (≥98%, analytical grade), chromatography-grade methanol (≥99.9%), and chromatography-grade acetonitrile (≥99.9%) were purchased from Shanghai Meriel Chemical Technology Co., Ltd., Shanghai, China. Methanol (≥99.5%, analytical grade) and phosphoric acid (≥85%, analytical grade) were obtained from Chengdu Kelong Chemicals Co., Ltd., Chengdu, China. Hesperidin (≥98%, HPLC grade), eupatilin (≥98%, HPLC grade), luteolin (≥98%, HPLC grade), kaempferol (≥98%, HPLC grade), isorhamnetin (≥98%, HPLC grade) were purchased from Chengdu Pushi Biotechnology Co., Ltd., Chengdu, China.

DL-Malic acid (≥99%), sorbitol (≥98%), ethylene glycol (≥99%), xylitol (≥98%), n-hexanoic acid (≥99%), glycerol (≥99.5%), L-proline (≥99%), succinic acid

(≥99.5%), glycol ethanoate (≥98%), tartaric acid (≥99.5%), and L-lactic acid (≥90%) were all purchased from Tianjin Xishenshi Biochemical Technology Co., Ltd., Tianjin, China.

The macroporous adsorption resins D101 (polystyrene-based, non-polar), NKA-2 (polystyrene-based, weakly polar), HPD-100 (polystyrene-based, non-polar), HPD-750 (polystyrene-based, moderately polar), XAD-7HP (acrylic ester-based, moderately polar), FPX-66 (polystyrene-based, non-polar), AB-8 (polystyrene-based, weakly polar), ADS-17 (polystyrene-based, hydrogen-bonding), and ADS-8 (polystyrene-based, moderately polar) were all purchased from Tianjin Guangfu Fine Chemical Research Institute, Tianjin, China.

Vitamin C (≥99%, analytical grade), sodium salicylate (≥99.5%), 3% hydrogen peroxide (v/v), ferrous sulfate heptahydrate (≥99%), α-glucosidase from Saccharomyces cerevisiae (≥50 U/mg), acetylcholinesterase from Electrophorus electricus (≥200 U/mg), pig pancreatic lipase (≥30 U/mg), phosphate-buffered saline (PBS, 0.1 M, pH 7.4), and potassium persulfate (≥99%) were purchased from Shanghai Youye Biotechnology Co., Ltd., Shanghai, China. 4-Nitrophenyl palmitate (pNPP, ≥98%) was obtained from the same supplier. 1,1-Diphenyl-2-picrylhydrazyl (DPPH, ≥95%), 2,2'-azino-bis(3-ethylbenzothiazoline-6-sulfonic acid) diammonium salt (ABTS, ≥98%), p-nitrophenyl-α-D-glucopyranoside (PNPG, ≥98%), anhydrous sodium carbonate (≥99.5%), acetylthiocholine iodide (ATCI, ≥98%), tris(hydroxymethyl)aminomethane hydrochloride (Tris-HCl, ≥99%), acarbose (≥98%), simvastatin (≥98%), and tixocortol pivalate (≥98%) were all purchased from Shanghai

Aladdin Biochemical Technology Co., Ltd., Shanghai, China.

S1.2 Determination of TF from *A. argyi*

Accurately weigh the medicinal powder and TDES into a suitable container. Conduct ultrasonic extraction at a liquid to solid ratio of 30 mL/g, ultrasonic power of 320 W, extraction temperature of 60 ℃, and extraction time of 50 min using a KQ-400DE ultrasonic cleaner (Shenzhen Fangao Microelectronics Co., Ltd., Shenzhen, China). Following extraction, centrifuge the extracted liquid at 3500 rpm for 20 min using a low speed centrifuge (Anhui Zhongke Zhongjia Scientific Instruments Co., Ltd., Hefei, China) to separate the supernatant. Collect the supernatant to obtain the sample extract.For preparation of the reference solution, accurately weigh 5.0 mg quercetin standard and dissolve completely in 70% methanol. Transfer to a 50 mL volumetric flask and dilute to volume with 70% methanol to obtain a stock solution with a concentration of 0.1 mg/mL. Store in the dark.

TF content of *A. argyi* was determined using a modified NaNO2-Al(NO3)3-NaOH colorimetric method based on Dia et al [1]. with modifications. Briefly, 1 mL of sample was mixed with 70% methanol to 5 mL. Then 0.3 mL of 5% sodium nitrite was added and incubated for 6 min, followed by addition of 0.3 mL of 10% aluminum nitrate and incubation for another 6 min. Finally, 4 mL of 4% sodium hydroxide was added, the mixture was diluted to 10 mL with 70% methanol, and incubated in the dark for 15 min. For the extract samples, TDES was used as blank control. Absorbance was measured at 510 nm using a UV spectrophotometer (Hitachi, Tokyo, Japan). A standard curve was constructed using

quercetin (5 to 40 microgram/mL) with the linear regression equation $y = 0.0167x + 0.1509$ ($R^2 = 0.9992$).

We analyzed the flavonoid composition of the TDES extract using high-performance liquid chromatography (HPLC). An Elite EClassical 3100 system equipped with a SinoChrom ODS2 C18 column (4.6 × 250 mm, 5 μm) and a UV detector was used. The column temperature was maintained at 30 °C, and the injection volume was 20 μL. The mobile phase consisted of acetonitrile (A) and 0.2% phosphoric acid in water (B). The flow rate was set to 1.0 mL/min. Separation was achieved using the following gradient program: 0–10 min, 20%–30% A; 10–25 min, 30%–50% A; 25–30 min, 35%–40% A; 30–40 min, 40%–100% A; 40–45 min, 100%–20% A. All standard solutions and samples were filtered through a 0.45 μm nylon membrane prior to injection. Detection was performed at 350 nm over a 45-minute run time. Standards including naringin, luteolin, kaempferol, and eupatilin were used for identification by retention time matching.

For quantitative analysis, individual stock solutions of naringin, luteolin, kaempferol, and eupatilin were prepared by accurately weighing 5.0 mg of each reference standard and dissolving in 70% methanol to a final volume of 5 mL, yielding a concentration of 1.0 mg/mL. Working standard solutions were prepared by serial dilution with 70% methanol to obtain concentrations of 0.2, 0.4, 0.6, 0.8, and 1.0 mg/mL. Calibration curves were constructed by plotting the peak area (y) against the concentration (x, mg/mL). As summarized in Table S1, excellent linearity was achieved for all four analytes, with correlation coefficients (R^2) ranging from 0.9991

to 0.9997 over the linear range of 0.2–1.0 mg/mL. The limits of detection (LOD) and quantification (LOQ) were determined based on signal-to-noise ratios of 3 and 10, respectively, and are listed in Table S1.

The TDES extract for quantification was prepared by collecting the supernatant from the optimized extraction, removing the TDES components under vacuum concentration, reconstituting the residue with 70% methanol, and filtering through a 0.45 μm nylon membrane prior to injection.

Method validation was conducted following standard analytical protocols. Precision was evaluated by injecting a mixed standard solution six times consecutively. The relative standard deviations (RSDs) of peak areas were 0.59% for naringin, 0.64% for luteolin, 0.71% for kaempferol, and 1.13% for eupatilin. Stability was assessed by analyzing the same sample solution at 0, 2, 4, 8, 12, and 24 h, yielding RSDs of 0.42%, 0.33%, 0.25%, and 0.41%, respectively. Repeatability was determined by preparing six independent sample solutions from the same batch, with RSDs of 1.10%, 1.41%, 0.91%, and 1.34% for the four compounds. Recovery tests were performed using a standard addition method at three concentration levels (low, medium, high) with three replicates each (n=9). Known amounts of reference standards were spiked into a known quantity of the sample. As shown in Table S2, the mean recoveries ranged from 100.07% to 100.39%, with RSDs between 0.94% and 1.42%. All validation results confirm that the developed HPLC method is accurate, precise, stable, and reliable for the quantification of flavonoids in *A. argyi* TDES extracts.

S1.3 Preparation and screening of TDES

A total of eight binary deep eutectic solvents (BDESs) and seventeen ternary deep eutectic solvents (TDESs) were prepared via the heating–stirring method, and their detailed compositions are listed in Table S3 [2]. The mixtures were heated in a water bath at 80 °C with continuous stirring until stable, homogeneous, and transparent liquids were formed. Using the extraction yield of TF from *A. argyi* as the evaluation index, ultrasound-assisted extraction was performed on *A. argyi* powder under the following conditions: ultrasonic temperature of 50 °C, liquid-to-solid ratio of 30:1 (mL/g), ultrasonic power of 320 W, and molar ratio of 1:1:1, with an extraction duration of 40 min. Following extraction, 3500 rpm (corresponding RCF unknown) for 20 min at 25 °C, and the supernatants were collected. The absorbance of each sample was measured according to the TF content determination method, and the TF content was calculated to screen for the optimal TDES.

To further optimize the TDES, *A. argyi* powder was extracted under the following conditions: liquid-to-solid ratio of 30:1 (mL/g), temperature of 50 °C, time of 40 min, power of 320 W, and Glycerol-Levulinic acid-Xylitol (Gly-LevA-Xyl) as the extraction solvent. Different molar ratios of Gly-LevA-Xyl (1:2:2, 1:2:3, and 1:3:1, molar ratio) and various water contents (10%, 20%, 30%, 40%, and 50%, molar fraction) were systematically investigated.

S1.4 Artificial neural network (ANN) modeling and genetic algorithm (GA) Optimization

A single-hidden-layer feedforward backpropagation (BP) neural network with three input neurons and one output neuron was constructed using MATLAB. The input variables were the liquid-to-solid ratio, extraction time, and extraction temperature, while the output variable was the TF yield. The hidden layer comprised eight neurons. Network training was performed using the Levenberg–Marquardt algorithm (trainlm). The maximum number of training epochs was set to 2000, the target mean squared error was 1×10^{-6}, and the minimum performance gradient was 1×10^{-8}. To rigorously evaluate model performance and avoid overfitting, the 17 datasets were randomly partitioned into a training set (12 groups), a validation set (3 groups), and an independent test set (2 groups). The training set was used to optimize network weights, the validation set was used to monitor training and prevent overfitting, and the test set provided an unbiased final evaluation. Input and output data were normalized to the [0, 1] interval using the mapminmax function; predicted values were subsequently reverse-normalized for model performance evaluation [3].

The trained artificial neural network (ANN) model was subsequently employed as the fitness function, coupled with a genetic algorithm (GA) to optimize the TF yield. The variable search space was defined as follows: liquid-to-solid ratio, 20-40 mL/g; extraction time, 40-60 min; and extraction temperature, 50-70 °C. The GA parameters were configured as follows: population size of 50, maximum number of generations of 100, and function tolerance of 1×10^{-6}. Finally, the optimal extraction conditions were determined using the GA-ANN model [4].

S1.5 TDES System and Recovery of TF from *A. argyi*

S1.5.1 Screening of Macroporous Resins

Under the optimized extraction conditions (liquid-to-solid ratio of 30 mL/g, extraction temperature of 60 °C, ultrasonic power of 320 W, and extraction time of 50 min), the optimal TDES system was employed to extract *A. argyi*. After centrifugation, the supernatant was collected and stored at 4 °C for subsequent use.

Nine types of pre-treated macroporous adsorption resins (AB-8, HPD-100, D101, NKA-2, HPD-750, ADS-17, XAD-7HP, ADS-8 and FPX-66) were individually placed in conical flasks, and the extract was added to each flask for static adsorption. Upon reaching adsorption equilibrium, the filtrate was removed. The saturated resins were then desorbed with 70% (v/v) methanol under identical conditions. The adsorption rate (equation 1) and desorption rate (equation 2) were calculated as follows [5].

$$\text{Adsorption rate } (A)=\frac{(C_0V_0-C_1V_1)}{C_0V_0}\times 100\% \qquad (1)$$

$$\text{Desorption rate } (D)=\frac{C_2V_2}{(C_0V_0-C_1V_1)}\times 100\% \qquad (2)$$

C_0 and C_1 denote the TF concentrations (mg/mL) before and after adsorption, respectively; C_2 denotes the TF concentration (mg/mL) in the desorption eluent. V_0, V_1, and V_2 represent the volumes (mL) of the extraction solution, post-adsorption filtrate, and desorption eluent, respectively.

S1.5.2 Static adsorption kinetics

Pre-treated XAD-7HP macroporous resin was added to a conical flask, followed by the addition of 30 mL of the extraction solution. Aliquots of the solution were withdrawn at predetermined time intervals (0-180 min), and the residual TF content was determined. The TF content was quantified by the $NaNO_2$-$Al(NO_3)_3$-NaOH colorimetric method, with absorbance measured at 510 nm using a UV-visible spectrophotometer. The adsorption rate of XAD-7HP at time *t* was calculated according to equation (3). The static adsorption curve was plotted with adsorption time as the abscissa and adsorption rate as the ordinate [5].

$$\text{Recovery rate } (R)=\frac{C_2V_2}{C_0V_0}\times100\% \qquad (3)$$

C_0 and C_2 denote the TF concentrations (mg/mL) of the extract before adsorption and the eluent after desorption, respectively; V_0 and V_2 represent the volumes (mL) of the extract added and the eluent, respectively.

S1.5.3 Determination of optimal eluent concentration

Following static adsorption of 10 mL of the extract onto 3.0 g of pre-treated XAD-7HP resin for 2 h, the effects of different methanol and ethanol concentrations (50%, 70%, and 100%, v/v) on the desorption efficiency of TF were investigated. The recovery rate was calculated according to equation (3):

S1.5.4 Evaluation of DES recycling performance

Pre-treated XAD-7HP macroporous resin was added to the ternary DES extraction solution. Static adsorption was performed at 25 °C and 100 rpm for 2 h.

The mixture was then filtered, and the filtrate was concentrated under vacuum to obtain the recovered DES. The recovered DES was subsequently employed to extract TF from A. argyi under the optimal extraction conditions. This cycle was repeated until the relative extraction yield fell below 80%. The recovery degree (R.D) and relative extraction rate (R.E) were calculated according to the following equations [6].

$$R.D=\frac{V_r}{V_n}\times 100\% \qquad (4)$$

$$R.E=\frac{E_r}{E_n}\times 100\% \qquad (5)$$

In the equations, *Vr* and *Vn* denote the volumes (mL) of recycled DES and freshly prepared DES, respectively; *Er* and *En* denote the TF extraction yields (mg/g) obtained with recycled DES and freshly prepared DES, respectively.

S1.6 Molecular dynamics (MD) simulations

The initial three-dimensional structures of eupatilin and solvent molecules (glycerol, xylitol, levulinic acid, and 30% water) were constructed and geometrically optimized using Discovery Studio 2024. Quantum-chemical geometry optimization was performed at the B3LYP/6-31G(d) level (ORCA 5.0). Single-point electrostatic potential (ESP) calculations were carried out at the HF/6-31G(d) level, and restrained electrostatic potential (RESP) atomic partial charges were fitted using Multiwfn (v3.8). Molecular topology, bonded and non-bonded parameters were generated under the GAFF2 force field via Sobtop (v1.0). Each system was solvated in a cubic periodic box (~7 × 7 × 7 nm^3) with TIP3P water, ensuring a minimum solute–wall distance of 1.0 nm.

Energy minimization: Steepest-descent minimization (max 50,000 steps, emtol = 1000 $kJ \cdot mol^{-1} \cdot nm^{-1}$, initial step 0.01 nm) was applied to eliminate atomic overlaps. Non-bonded interactions used Verlet neighbor-list (update every 10 steps, rlist = 1.0 nm); electrostatics: PME (rcoulomb = 1.0 nm); van der Waals: cutoff (rvdw = 1.0 nm), 3D periodic boundary conditions.

Pre-equilibration: (1) NVT (100 ps): V-rescale thermostat ($\tau = 0.1$ ps, T = 298.15 K); H-bonds constrained by LINCS; PME real-space cutoff = 0.9 nm. (2) NPT (100 ps): Parrinello–Rahman barostat ($\tau = 2$ ps, P = 1 bar, compressibility = 4.5×10^{-5} bar^{-1}); temperature settings identical to NVT.

Production MD: 20 ns NPT production (2 fs timestep, leap-frog integrator, total 10,000,000 steps). T = 298.15 K (V-rescale), P = 1 bar (Parrinello–Rahman). H-bonds constrained by LINCS. PME electrostatics (real-space cutoff = 0.9 nm, grid spacing 0.16 nm, order 4); vdW truncated at 0.9 nm with long-range dispersion correction (DispCorr = EnerPres). Coordinates saved every 10 ps.

H-bond analysis: GROMACS gmx hbond: D–A distance ≤ 3.5 Å, D–H–A angle ≥ 120°. Continuous lifetime distribution computed over the full 20 ns; τ obtained by fitting to $C(t) = \exp(-t/\tau)$. Three independent runs with different initial velocity seeds were averaged; mean τ reported in Section 3.10 [7,8].

S1.7 In vitro antioxidation

S1.7.1 DPPH radical scavenging activity

According to the method of Yoo et al. [9,10], the TF and Vc reference solution was prepared into different concentrations (0.05-0.8 mg/mL). Precisely weigh 1.97

mg of DPPH powder, add anhydrous ethanol to make the volume 25 mL, store it in the dark and keep it ready. This is the DPPH free radical working solution. Take several test tubes, respectively add 2 ml of different concentrations of TF solution and Vc reference solution, then add 2 mL of the prepared DPPH free radical working solution to each, mix the two solutions evenly, and place them in the dark at 25 °C for 30 min. Measure the absorbance of the samples at 517 nm using a UV-visible spectrophotometer. Calculate the scavenging rate of samples at different concentrations according to equation (6):

$$\text{Scavenging rate (\%)}=\frac{A_0-A_X}{A_0}\times 100\% \quad (6)$$

Where A_0 is the absorbance of the blank control group, and A_x is the absorbance of the sample.

S1.7.2 ABTS radical scavenging activity

Based on the method of Yoo et al. [10] with minor modifications, the ABTS stock solution was prepared by mixing 7 mmol/L ABTS solution and 5 mmol/L potassium persulfate at a volume ratio of 1:1. The mixture was incubated in the dark at 25 °C (room temperature) for 14 h to allow complete radical generation. The ABTS working solution was prepared by diluting 5 mL of the stock solution with anhydrous ethanol (1:20, v/v). Aliquots (2 mL) of the ABTS working solution were mixed with 2 mL of TF solution or Vc reference solution at various concentrations (0.05-0.80 mg/mL) in separate test tubes. The mixtures were incubated in the dark at 25 °C for 6 min, and the absorbance of each sample was measured at 734 nm using a UV–visible spectrophotometer. The scavenging rate was calculated according to equation (6).

S1.7.3 Hydroxyl radical scavenging activity

Based on the method proposed by S. Chen et al. [9,11] with modifications, the hydroxyl radical scavenging activity was investigated using the Fenton reaction system. The experimental groups were configured as follows: Group A_3 contained 50 μL each of TF solution at various concentrations (0.20-1.20 mg/mL), 9 mmol/L ferrous sulfate, 9 mmol/L salicylic acid–ethanol solution, and 8.8 mmol/L hydrogen peroxide; Group A_4 contained 50 μL each of TF solution at various concentrations (0.20-1.20 mg/mL), 9 mmol/L ferrous sulfate, 9 mmol/L salicylic acid–ethanol solution, and deionized water; Group A_5 (blank control) contained 50 μL each of 9 mmol/L ferrous sulfate, 9 mmol/L salicylic acid–ethanol solution, deionized water, and 8.8 mmol/L hydrogen peroxide. The mixtures were incubated at 37 °C for 30 min, and the absorbance of each sample was measured at 510 nm using a UV–visible spectrophotometer. The hydroxyl radical scavenging rate was calculated according to equation (7):

$$\text{scavenging rate } (\%)=(1-\frac{A_3\text{-}A_4}{A_5})\times 100\% \qquad (7)$$

Where, A_5 is the absorbance of the blank control group, A_3 is the absorbance of the TF solution, and A_4 is the absorbance of the TF solution without H_2O_2 added.

S1.8 Antioxidant molecule interaction study

The 3D structure of the eupatilin molecule was constructed and geometrically optimized. Eupatilin, the predominant flavonoid constituent in *A. argyi* TF, was selected as the representative compound for molecular docking simulation. The molecular structure was processed using AutoDockTools 4 [12]. Global docking

(flexible docking) of eupatilin was subsequently performed against the ABTS and DPPH molecules in two separate groups using AutoDock Vina 1.2.7 [13,14]. In this docking protocol, the ABTS and DPPH molecules were individually treated as receptors, while eupatilin served as the ligand. The grid spacing parameter in AutoDock Vina was set to the default value of 0.375 Å, and the exhaustiveness parameter was set to the default value of 8. The processed receptor and ligand PDBQT files were input into AutoDock Vina, and molecular docking was executed according to the aforementioned settings. Upon completion of each docking group, the resulting conformations were ranked according to their docking scores, and the optimal structure was selected according to the lowest binding free energy for subsequent analysis.

S1.9 In vitro enzyme inhibitory activity

The biological activities of TF from *A. argyi* in regulating glucose and lipid metabolism and neuroprotection were investigated. The α-glucosidase inhibitory activity of TF was evaluated using the method described by Dej-adisai et al. [15,16], with slight modifications. The concentrations of TF tested ranged from 5 to 150 μg/mL. Acarbose was employed as the positive control. The enzymatic reaction was conducted in 0.1 M PBS buffer at 37 °C, using PNPG as the substrate. The final concentrations of enzyme and PNPG substrate were 0.00011 mM and 3.921 mM, respectively. Following the enzymatic reaction, the absorbance was measured at 412 nm, and the inhibition rate was calculated according to equation (8).

$$\text{Inhibition rate (\%)}=\left(1-\frac{A_s-A_b}{A_c}\right)\times 100\% \qquad (8)$$

where A_S is the absorbance of the sample group; A_b is the absorbance of the sample blank group; A_C is the absorbance of the control group.

The acetylcholinesterase (AChE) inhibitory activity of TF was assessed using the method reported by Yang et al. [8], with minor adjustments. The concentrations of Artemisia TF tested ranged from 0.25 to 3 mg/mL. Tacrine was used as the positive control. After the catalytic reaction of porcine pancreatic lipase and chromogenic substrate, the absorbance was measured at 412 nm. and the enzyme activity inhibition rate was calculated according to equation (9).

$$\text{Inhibition rate (\%)}=\left(\frac{(A_3-A_2)-(A_1-A_4)}{A_3-A_2}\right)\times 100\% \qquad (9)$$

Where, A_1 is the absorbance after adding the sample and reacting; A_2 is the absorbance after no sample and 4-nitrophenyl acetate (ATCI) addition but reaction; A3 is the absorbance after no sample but reacting; A_4 is the absorbance after adding the sample but no ATCI addition for reaction.

The porcine pancreatic lipase inhibitory activity of TF was evaluated using a modified method as described in reference [8] The concentrations of TF tested ranged from 0.05 to 2.0 mg/mL. Simvastatin was employed as the positive control. Following the porcine pancreatic lipase inhibition reaction, the absorbance was measured at 405 nm, and the inhibition rate was calculated using equation (10).

$$\text{Inhibition rate (\%)}=\left(1-\frac{A_x-A_5}{A_0-A_6}\right)\times 100\% \qquad (10)$$

Where, A_X represents the absorbance of the sample solution, A_0 represents the absorbance of the control, A_5 represents the absorbance of the solution without enzyme, and A_6 represents the absorbance of the solution without the sample and the

enzyme.

S2 Results and discussion

S2.1 TDES system and recovery of TF from *A. argyi*

S2.1.1 Screening of macroporous resin

Using the adsorption rate and desorption rate of TF as the evaluation indicators, we screened nine types of macroporous resins, including AB-8, HPD-100, D101, NKA-2, HPD750, ADS-17, XAD-7HP, ADS-8 and FPX-66. The results are shown in Figure 9A. Compared with other macroporous adsorption resins, XAD-7HP has the best adsorption and desorption effects on TF in *A. argyi.* The adsorption rate and desorption rate are 81.3% and 70.4% respectively. Therefore, XAD-7HP was selected for enriching TF in *A. argyi*.

S2.1.2 Static adsorption kinetics

When evaluating resin performance, adsorption and desorption capacities are not the sole criteria. Other factors affecting the adsorption efficiency, such as the adsorption rate, should also be considered. The static adsorption curve of TF from *A. argyi* by XAD-7HP macroporous resin is shown in Figure. 9B. Within the initial 30 min, the adsorption rate was rapid. After extending the adsorption time to 2 h, the adsorption of TF from *A. argyi* by the resin was essentially complete, with an adsorption efficiency of approximately 74.0% (100% refers to the initial total flavonoid amount in the loading solution). The rapid attainment of adsorption equilibrium by XAD-7HP indicates that this macroporous resin exhibits favorable adsorption kinetics for *A. argyi* TF, enabling relatively fast equilibration.

S2.1.3 Determination of elution alcohol concentration

Taking the recovery rate as the indicator, the effects of different concentrations of methanol and ethanol on the TF recovery rate were investigated. The results are shown in Table S5. When the ethanol elution concentration was 70%, the recovery rate of TF by XAD-7HP was the best at 69.55 ± 1.63%. Therefore, an ethanol concentration of 70% was selected for the elution of TF.

S2.1.4 Examination of the recycling and repurposing frequency of TDES

In this study, the TF in the TDES extract were adsorbed by the macroporous resin XAD-7HP, and DES could be recovered and reused. As shown in Table S6, the recovered Gly-LevA-Xly could be recycled 3 times. The recovery rate of TDES was 66.7 ± 3.51%, and the extraction rate of TF by the recovered TDES was 78.0 ± 2.16%. This means that through appropriate recovery processing, we can not only effectively extract TF from *A. argyi*, but also recover most of the used TDES, thereby achieving the recycling of resources.

References:

1. Dai, H.; Hu, M.; Zhu, N.; Song, L.; Hong, H. Extraction of Flavonoids from *Artemisia Argyi* Using Deep Eutectic Solvents and Its Antioxidant Activities. *J. Mol. Liq.* **2025**, *426*, 127236, doi:10.1016/j.molliq.2025.127236.
2. Guo, X.; Liu, P.; Zhang, M.; Song, M.; Shi, M.; Gao, H.; Cui, J. Extraction of Lycium Barbarum Pigment by Surfactant-Assisted Deep Eutectic Solvents and Its Antioxidant Activity. *Appl. Biochem. Biotechnol.* **2026**, doi:10.1007/s12010-026-05716-8.
3. Jiang, D.-Z.; Yu, D.-P.; Zeng, M.; Liu, W.-B.; Li, D.-L.; Liu, K.-Y. Optimization of Ultrasonic-Assisted Extraction of Total Flavonoids from Oxalis Corniculata by a Hybrid Response Surface Methodology-Artificial Neural Network-Genetic Algorithm (RSM-ANN-GA) Approach, Coupled with an Assessment of Antioxidant Activities. *RSC Adv.* **2024**, *14*, 39069–39080, doi:10.1039/D4RA05077K.
4. Ran, H.; Zhang, X.; Long, J.; Wang, Z.; Zhuang, S.; Zhang, X.; Jiang, Y.; Zhang, J.; Wang, G. Ultrasound-Assisted Extraction of Naringin from Exocarpium Citri Grandis Using a Novel Ternary Natural Deep Eutectic Solvent Based on Glycerol: Process Optimization

Using ANN-GA, Extraction Mechanism and Biological Activity. *Ultrason. Sonochem.* **2025**, *121*, doi:10.1016/j.ultsonch.2025.107551.

5. Li, J.; Mu, M.; Xu, R.; Chen, B.; Sun, X.; Dai, C. Environmental Sustainability of Levulinic Acid Donor-Based Deep Eutectic Solvents for Dichloromethane Absorption. *Acs Sustain. Chem. Eng.* **2025**, *13*, 20534–20544, doi:10.1021/acssuschemeng.5c09627.
6. Li, G.; Lei, J.; Li, S.; Jiang, Y.; Zhang, F.; Song, C.; Xiao, S.; Fu, S.; Zhou, J.; Wu, F.; et al. Extraction of Flavonoids from Citri Reticulatae Pericarpium Viride Using a Deep Eutectic Solvent. *Rsc Adv.* **2022**, *12*, 26975–26988, doi:10.1039/d2ra04276b.
7. Su, Y.; Chen, P.; Wen, Y.; Yang, J.; Chen, Y.; Zhu, S.; Zhan, S.; Zheng, S. Deciphering the Molecular Networks of 3-Methylcholanthrene-Induced Clear Cell Renal Cell Carcinoma through Multi-Omics Integration. *Sci. Rep.* **2026**, *16*, 4411, doi:10.1038/s41598-025-34526-x.
8. Yang, X. In Vivo and Bioinformatics Evaluation of Nine Traditional Chinese Medicine Compounds Targeting Acetylcholinesterase and Butyrylcholinesterase Enzymes in Alzheimer's Disease. *IJ Pharmaceutical Research.* **2025**, *24*, e159760, doi:10.5812/ijpr-159760.
9. Chen, S.; Liu, D.; Zhang, P.; Zhu, P.; Tan, W.; Liu, L. Effects of Drying Methods on the Chemical Composition and Antioxidant Activity of Metapanax Delavayi by Integrating UHPLC-Q-TOF-MS and GC–MS/MS Based Untargeted Metabolomics. *Food Chem. X* **2026**, *34*, 103664, doi:10.1016/j.fochx.2026.103664.
10. Yoo, J.; Lee, S.; Song, S.; Kim, C. Functional Characteristics of Peptides from Whey Proteins Fermented with Lactic Acid Bacteria Isolated from Dongchimi. *Food Sci. Anim. Resour.* **2026**, *46*, 50, doi:10.1007/s44463-025-00053-6.
11. Chen, L.; Yang, J.; Wang, Y.; Xiao, X.; Zhang, H.; Hou, X.; Liu, D.; Du, H. *Artemisia Argyi* Folium as a Traditional and Safe Dietary Additive Ameliorates Metabolic Syndrome via Gut Microbiota and Its Metabolites. *Food Res. Int.* **2026**, *223*, 117830, doi:10.1016/j.foodres.2025.117830.
12. Eberhardt, J.; Santos-Martins, D.; Tillack, A.F.; Forli, S. AutoDock Vina 1.2.0: New Docking Methods, Expanded Force Field, and Python Bindings. *J. Chem. Inf. Model.* **2021**, *61*, 3891–3898, doi:10.1021/acs.jcim.1c00203.
13. Rampogu, S.; Balasubramaniyam, T.; Lee, J.-H. Curcumin Chalcone Derivatives Database (CCDD): A Python Framework for Natural Compound Derivatives Database. *PeerJ.* **2023**, *11*, e15885, doi:10.7717/peerj.15885.
14. Zhu, D.; Zheng, H.; Xie, X.; Zhang, B. A Novel Antioxidant Peptide from Black Soybean [Glycine Max (L.) Merr.] with Verified MPO Interaction and Multi-Scale Antioxidant Activity. *Front. Nutr.* **2025**, *12*, doi:10.3389/fnut.2025.1649684.
15. Bindra, S.; Mostafa, E.M.; Abdelgawad, M.A.; Selim, S.; Kumar, S.; Mathew, B. Synthetic Strategies and Medicinal Chemistry Perspectives of Dual Acting Carbonic Anhydrase Modulators with Monoamine Oxidase and Cholinesterase Inhibitors. *RSC Med. Chem. 16*, 1532–1549, doi:10.1039/d4md00837e.
16. Dej-adisai, S.; Sakulkeo, O.; Wattanapiromsakul, C.; Pitakbut, T. Flavonoid Constituents and Alpha-Glucosidase Inhibition of Solanum Stramonifolium Jacq. Inflorescence with In Vitro and In Silico Studies. *Molecules.* **2022**, *27*, doi:10.3390/molecules27238189.

Table S1 The calibration curves, linear ranges, LODs and LOQs

component	calibration curves	linear range (mg/mL)	R^2	LOD (mg/mL)	LOQ (mg/mL)
Naringin	y = 5668.7x - 421.29	0.2-1	0.9994	0.601	0.203
Luteolin	y = 72616x - 2204.5	0.2-1	0.9992	0.028	0.094
Kaempferol	y = 43434x - 174.02	0.2-1	0.9997	0.047	0.157
Eupatilin	y = 51752x + 117.21	0.2-1	0.9991	0.040	0.132

Table S2 Results of recovery tests for the four flavonoids (n=9)

Component	No.	Original amount (mg)	Spiked amount (mg)	Found amount (mg)	Recovery (%)	Mean (%)	RSD (%)
Naringin	1	0.3156	0.2525	0.5654	99.17	100.34	1.42
	2	0.3156	0.2525	0.5714	101.35		
	3	0.3156	0.2525	0.5763	103.08		
	4	0.3659	0.3657	0.7271	98.96		
	5	0.3659	0.3657	0.7438	103.36		
	6	0.3659	0.3657	0.7362	101.26		
	7	0.3396	0.4087	0.7439	98.90		
	8	0.3396	0.4087	0.7510	100.66		
	9	0.3396	0.4087	0.7598	102.81		
Luteolin	1	0.1629	0.1303	0.2915	98.78	100.07	1.21
	2	0.1629	0.1303	0.2938	100.54		
	3	0.1629	0.1303	0.2961	102.30		
	4	0.1629	0.1629	0.3238	98.72		
	5	0.1629	0.1629	0.3284	101.51		
	6	0.1629	0.1629	0.3261	100.12		
	7	0.1629	0.1947	0.3552	98.70		
	8	0.1629	0.1947	0.3598	101.08		
	9	0.1629	0.1947	0.3621	102.44		
Kaempferol	1	0.0841	0.0673	0.1508	98.97	100.12	1.08

Component	No.	Original amount (mg)	Spiked amount (mg)	Found amount (mg)	Recovery (%)	Mean (%)	RSD (%)
	2	0.0841	0.0673	0.1519	100.62		
	3	0.0841	0.0673	0.1526	101.66		
	4	0.0841	0.0841	0.1675	99.10		
	5	0.0841	0.0841	0.1688	100.66		
	6	0.0841	0.0841	0.1682	99.94		
	7	0.0841	0.1009	0.1837	98.63		
	8	0.0841	0.1009	0.1859	100.81		
	9	0.0841	0.1009	0.1866	101.55		
Eupatilin	1	0.2532	0.2026	0.4553	99.85	100.39	0.94
	2	0.2532	0.2026	0.4564	100.38		
	3	0.2532	0.2026	0.4587	101.44		
	4	0.2532	0.2532	0.5051	99.57		
	5	0.2532	0.2532	0.5073	100.42		
	6	0.2532	0.2532	0.5062	99.97		
	7	0.2532	0.3038	0.5568	99.87		
	8	0.2532	0.3038	0.5591	100.64		
	9	0.2532	0.3038	0.5614	101.41		

Table S3 List of prepared TDESs

No.	Components	Abbreviation	Mole ratio	Water content (w/w, %)
DES-1	Sorbitol-levulinic acid	Sor-LevA	1：1	20
DES-2	Sorbitol-malic acid	Sor-Mal	1：1	20
DES-3	Propylene glycol-levulinic acid	PG-Lev	1：1	20
DES-4	Ethylene glycol-xylitol	EG-Xyl	1：1	20
DES-5	Propylene glycol- hexanoic acid	PG-Hex	1：1	20
DES-6	Glycerol-L-proline	Gly-Pro	1：1	20
DES-7	Glycerol-malic acid	Gly-Mal	1：1	20
DES-8	Glycerol-levulinic acid	Gly-LevA	1：1	20
DES-9	Sorbitol-levulinic acid-malonic acid	Sor-LevA-Mal	1：1：1	20
DES-10	Sorbitol-levulinic acid-L-proline	Sor-LevA-Pro	1：1：1	20
DES-11	Sorbitol-levulinic acid-glycolic acid	Sor-LevA-Glyc	1：1：1	20
DES-12	Propylene glycol-levulinic acid-glycolic acid	PG-LevA-Glyc	1：1：1	20
DES-13	Ethylene glycol-levulinic acid-xylitol	EG-LevA-Xyl	1：1：1	20
DES-14	Ethylene glycol-levulinic acid-tartaric acid	EG-LevA-Tart	1：1：1	20
DES-15	Ethylene glycol-levulinic acid-malic acid	EG-LevA-Mal	1：1：1	20
DES-16	Ethylene glycol-levulinic acid-malonic acid	EG-LevA-Maln	1：1：1	20
DES-17	Ethylene glycol-levulinic acid-glycolic acid	EG-LevA-Glyc	1：1：1	20
DES-18	Glycerol-levulinic acid-hexanoic acid	Gly-LevA-Hex	1：1：1	20
DES-19	Glycerol-levulinic acid-xylitol	Gly-LevA-Xyl	1：1：1	20

No.	Components	Abbreviation	Mole ratio	Water content (w/w, %)
DES-20	Glycerol-levulinic acid-tartaric acid	Gly-LevA-Tart	1：1：1	20
DES-21	Glycerol-levulinic acid-malic acid	Gly-LevA-Mal	1：1：1	20
DES-22	Glycerol-levulinic acid-malonic acid	Gly-LevA-Maln	1：1：1	20
DES-23	Glycerol-levulinic acid-glycolic acid	Gly-LevA-Glyc	1：1：1	20
DES-24	Xylitol-levulinic acid-malic acid	Xyl-LevA-Mal	1：1：1	20
DES-25	Xylitol-levulinic acid-lactic acid	Xyl-LevA-Lac	1：1：1	20

Table S4 Box-Behnken design table of factor levels

Factor	Coding level		
	-1	0	1
A: liquid-to-solid ratio (mL/g)	20	30	40
B: Extraction time (min)	40	50	60
C: Extraction temperature (℃)	50	60	70

Table S5 Box-Behnken Experimental Results

Run	A: liquid-to-solid ratio (mL/g)	B: Extraction time (min)	C: Extraction temperature (℃)	TF extraction yield (mg/g)
1	20	40	60	81.71
2	40	40	60	83.93
3	20	60	60	72.87
4	40	60	60	89.64
5	20	50	50	75.19
6	40	50	50	89.90
7	20	50	70	75.32
8	40	50	70	83.91
9	30	40	50	80.32
10	30	60	50	92.53
11	30	40	70	87.64
12	30	60	70	87.20
13	30	50	60	102.72
14	30	50	60	107.13
15	30	50	60	105.32
16	30	50	60	103.91
17	30	50	60	107.76

Table S6 Analysis of Variance for the Regression Equation

SoUce	Sum of	df	*F-value*	*p-value*	R^2	R^2	Significant
Model	2129.07	9	29.64	< 0.0001	0.9744	0.9416	significant
A	224.72	1	28.16	0.0011			
B	9.24	1	1.16	0.3175			
C:	1.80	1	0.2262	0.6489			
AB	53.29	1	6.68	0.0363			
AC	9.61	1	1.20	0.3088			
BC	39.69	1	4.97	0.0610			
A^2	896.90	1	112.39	< 0.0001			
B^2	322.00	1	40.35	0.0004			
C^2	395.76	1	49.59	0.0002			
Residual	55.86	7					
LevAck of Fit	38.15	3	2.87	0.1672			Not significant
PUe Error	17.71	4					
Cor Total	2184.93	16					

Table S7 Investigation of elution concentration for methanol and ethanol

Serial Number	Decontamination solvent	TF recovery rate (%)
1	50% methanol	40.63±2.76
2	70% methanol	66.38±1.95
3	100% methanol	65.80±2.06
4	50% ethanol	57.06±3.66
5	70% ethanol	69.55±1.63
6	100% ethanol	59.01±1.82

Table S8 Recovery of TDES

Recycling frequency	Recycling rate (%)	Relative extraction rate (%)
1	96.83±1.82	100.00
2	85.18±2.02	88.40±2.97
3	88.23±2.07	84.53±3.76
4	66.66±3.51	78.01±2.16

Table S9. Calculated single-point energies and binding energies of solvent–cellobiose complexes at the DFT level

Species / Complex	Single-Point Energy (a.u.)	Binding Energy (a.u.)	Binding Energy (kcal/mol)
DES (Gly:LevA:Xyl = 1:1:1)	−1339.7	—	—
XWET (Cellobiose)	−1297.9	—	—
DES–XWET (Complex)	−2637.7	−0.0298	−18.67
eth (Ethanol)	−155.0	—	—
eth–XWET (Complex)	−1763.1	−0.0394	−24.67
GY (Glycerol)	−344.8	—	—
MTC (Levulinic acid)	−573.9	—	—
YXBS (Xylitol)	−421.0	—	—

Table footnote: Binding energies (ΔE) were calculated as ΔE = E(Complex) − [E(Solvent) + E(Cellobiose)]; "—" indicates monomers without complex formation. Negative values denote exothermic, stabilizing interactions.

Table S10. Molecular docking results

#	Receptor	Ligand	Binding Energy (kcal/mol)
1	ABTS	Eupatilin	-4.515
2	DPPH	Eupatilin	-4.083

Table S11.Binding energies of eupatilin and allopurinol to xanthine oxidase.

#	Receptor	Ligand	Binding Energy (kcal/mol)
1	xanthine oxidase	Eupatilin	−7.778
2	xanthine oxidase	Allopurinol	−6.86

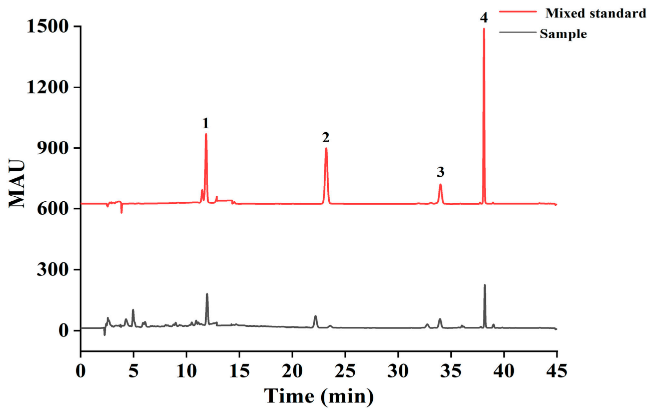

Figure S1. HPLC chromatograms of TF sample and mixed standard (1: Naringin, 2: Luteolin, 3: Kaempferol, 4: Eupatilin)

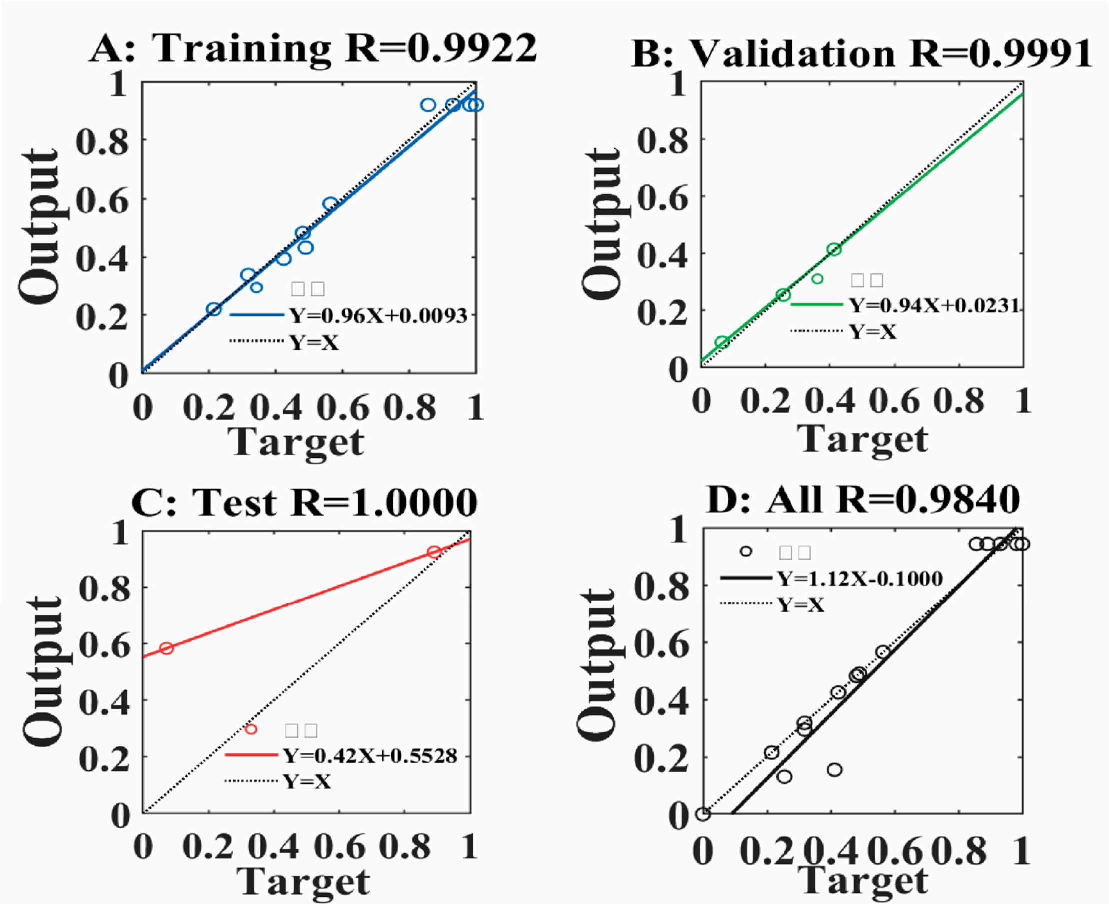

Figure S2 Regression plots for training, validation, test, and all data Sets.

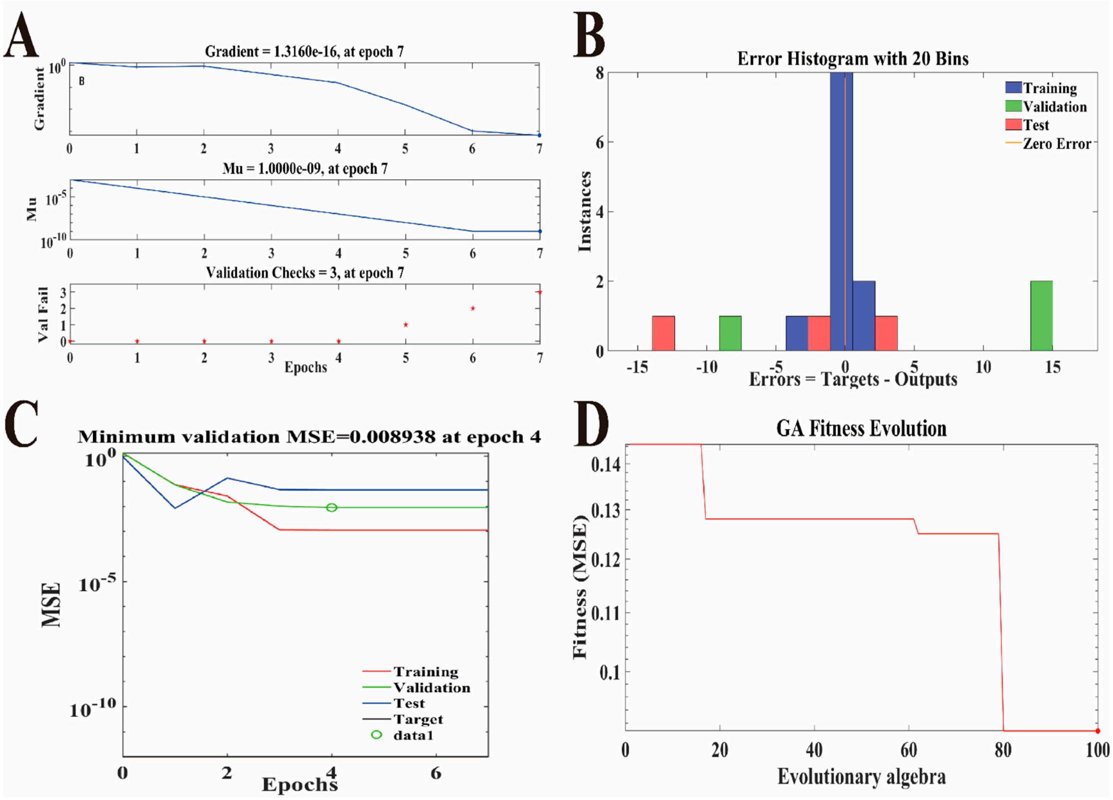

Figure S3 (A) Regression analysis of TF yield prediction for training, validation, and test sets; (B) Error histogram of prediction errors for all datasets; (C) MSE convergence curve of the ANN during training; (D) GA fitness evolution curve over generations.

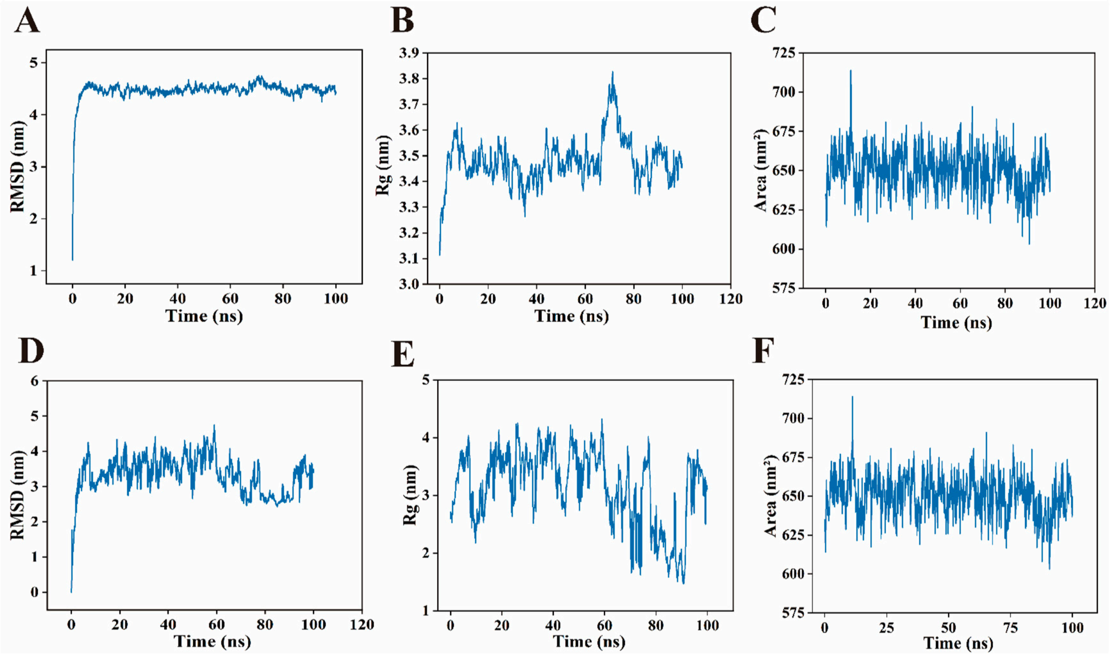

Figure S4 Molecular dynamics simulation profiles over time. (A) RMSD curve of the TDES system over time; (B) Radius of gyration (Rg) curve of the TDES system over time; (C) Solvent accessible surface area (SASA) curve of the TDES system over time; (D) RMSD curve of eupatilin over time; (E) Radius of gyration (Rg) curve of eupatilin over time; (F) Solvent accessible surface area (SASA) curve of eupatilin over time.
